# Comparative transcriptome analyses of fruit development among pears, peaches, and strawberries provide new insights into single sigmoid patterns

**DOI:** 10.1186/s12870-020-2317-6

**Published:** 2020-03-06

**Authors:** Mao-Song Pei, Su-Hao Cao, Lei Wu, Guo-Ming Wang, Zhi-Hua Xie, Chao Gu, Shao-Ling Zhang

**Affiliations:** Centre of Pear Engineering Technology Research, State Key Laboratory of Crop Genetics and Germplasm Enhancement, Nanjing Agricultural University, Nanjing, 210095 China

**Keywords:** Pear, Peach, Strawberry, Single sigmoid pattern, QTL, Gene regulation network

## Abstract

**Background:**

Pear fruit exhibit a single sigmoid pattern during development, while peach and strawberry fruits exhibit a double sigmoid pattern. However, little is known about the differences between these two patterns.

**Results:**

In this study, fruit weights were measured and paraffin sections were made from fruitlet to maturated pear, peach, and strawberry samples. Results revealed that both single and double sigmoid patterns resulted from cell expansion, but not cell division. Comparative transcriptome analyses were conducted among pear, peach, and strawberry fruits at five fruit enlargement stages. Comparing the genes involved in these intervals among peaches and strawberries, 836 genes were found to be associated with all three fruit enlargement stages in pears (Model I). Of these genes, 25 were located within the quantitative trait locus (QTL) regions related to fruit weight and 90 were involved in cell development. Moreover, 649 genes were associated with the middle enlargement stage, but not early or late enlargement in pears (Model II). Additionally, 22 genes were located within the QTL regions related to fruit weight and 63 were involved in cell development. Lastly, dual-luciferase assays revealed that the screened *bHLH* transcription factors induced the expression of cell expansion-related genes, suggesting that the two models explain the single sigmoid pattern.

**Conclusions:**

Single sigmoid patterns are coordinately mediated by Models I and II, thus, a potential gene regulation network for the single sigmoid pattern was proposed. These results enhance our understanding of the molecular regulation of fruit size in *Rosaceae*.

## Background

Within the *Rosaceae*, different fleshy fruit types showed two different fruit growth patterns*.* Pome fruits such as apple and pear exhibit a single sigmoid pattern in which fruits undergo extensive cell division during the first few weeks immediately following fertilization, after which almost all growth is due to cell enlargement [1–4]. Stone fruits such as late-maturing peach exhibit a double sigmoid pattern in which two rapid-growth stages are separated by a slow-growth stage [2, 5, 6]. Interestingly, strawberry fruits exhibit either a single or double sigmoid pattern in different varieties [7–12]. These results indicate that fruit growth patterns are determined by other factors than by the type of fruit.

The distinction between single and double sigmoid patterns is whether a slow-growth period occurs during fruit enlargement. A previous study reported that cooperation between the velocity and duration of fruit swelling determines fruit size [13], which is determined by both cell number and size [14–16]. Currently, little is known about the genes that control fruit size, with the exception of genes involved in the cell cycle, cell wall metabolism, cytochrome, and ubiquitin [13, 17, 18]. Specifically, *cyclin*, *RNA polymerase II transcription*, and *mitogen-activated protein kinase kinase kinase* are involved in the cell cycle [19–21], *xyloglucan galactosyltransferase*, *glycosyltransferase*, *cellulose synthase*, *β-galactosidase*, and *microtubule-associated proteins* are associated with cell wall metabolism [22–25], and transcription factors, including *basic region/leucine zipper motif* (*bZIP*) [26], *NAM/ATAF1/2/CUC2* (*NAC*) [27], *v-myb avian myeloblastosis viral oncogene homolog* (*MYB*) [28], *basic/helix-loop-helix* (*bHLH*) [29], and *WRKY* [30], are components of the fruit size regulation network.

A combination of transcriptome sequencing and QTL is an effective method for screening candidate genes of specific traits and has been used in plants, including peaches, pears, and tomatoes [27, 28, 31, 32]. Currently, fruit size is known to be anchored by 28 QTLs distributed on 11 chromosomes in tomatoes [33, 34]. In pome, fruit weight, height, and width were individually anchored by 14, 3, and 4 QTLs, respectively, on 5 chromosomes and 9 scaffolds in pears [35–37], as well as anchored by 10, 7, and 10 QTLs, respectively, on 7 chromosomes in apples [38]. In drupe, fruit weight, height and width were individually anchored by 7, 6, and 12 QTLs, respectively, on all 8 chromosomes in peaches [39–41], and anchored by 6, 2, and 2 QTLs, respectively, on 4 chromosomes in sweet cherries [42, 43]. Moreover, fruit weight was anchored by 3 QTLs on Chr2 in strawberries [44, 45]. These reported QTL regions provide a good reference for screening candidate genes of agronomic traits.

Single and double sigmoid patterns have been reported in the last century in *Rosaceae* fruit species [2], but the difference between these patterns has not been explored at the molecular level until now. In *Rosaceae*, pear is a pome fruit and was selected for studies of single sigmoid pattern, while peach is a drupe fruit that presents a double sigmoid pattern and was selected as a control. Moreover, strawberry is an aggregate fruit that present sigmoid or double pattern and was selected as another control. The fruits in these three species were selected and performed for transcriptome sequencing to explore the difference between single and double patterns. First, developing and matured fruits were collected for measuring fruit weight and calculating cell number to clarify the periods of rapid enlargement and intervals in peaches and strawberries. Based on the completed genome projects of pears, peaches, and strawberries [46–48], transcriptome sequencing was conducted to screen for differentially expressed genes (DEGs) related to fruit enlargement in the three *Rosaceae* species. Validation of the candidate genes was conducted based on reported QTLs [35, 39, 44], functional annotation was also performed. Moreover, a dual-luciferase assay was conducted between the screened transcription factors and cell expansion-related genes. Finally, molecular regulation of the single sigmoid pattern was discussed. This is the first study to report on the difference between single and double sigmoid patterns at the molecular level. These results will enhance our understanding of the molecular regulation of fruit size in *Rosaceae*.

## Results

### Investigation of growth curves in pear, peach, and strawberry fruits

**Fig. 1:**
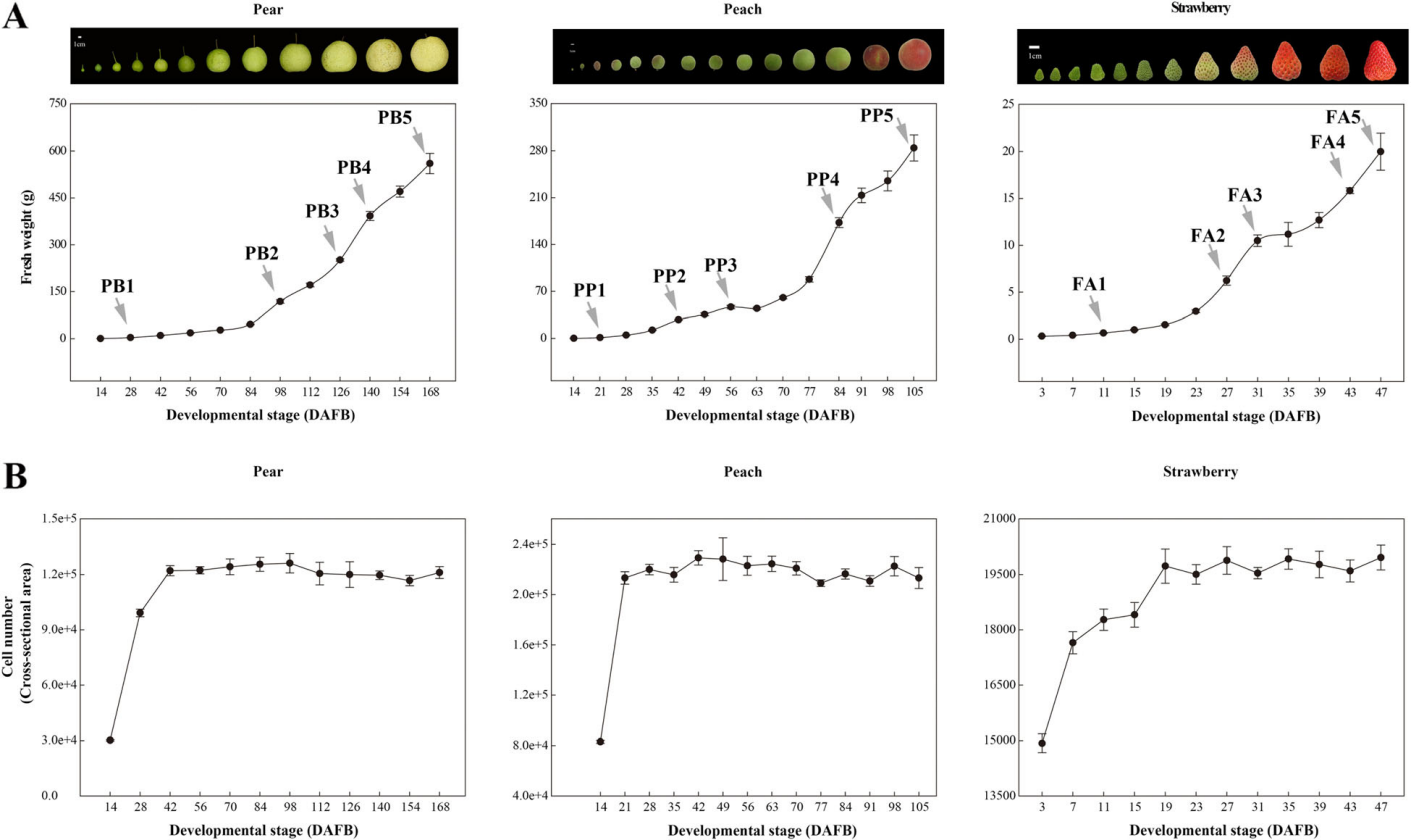
Measurements of fruit weight and cell number in pear, peach, and strawberry fruits. **a** Fresh weight (g) of pear, peach, and strawberry fruitlets to matured fruit samples. **b** Cell numbers of pear, peach, and strawberry fruitlets to matured fruits per fruit. Standard errors (SEs) were calculated from 15 biological replicates

**Fig. 2:**
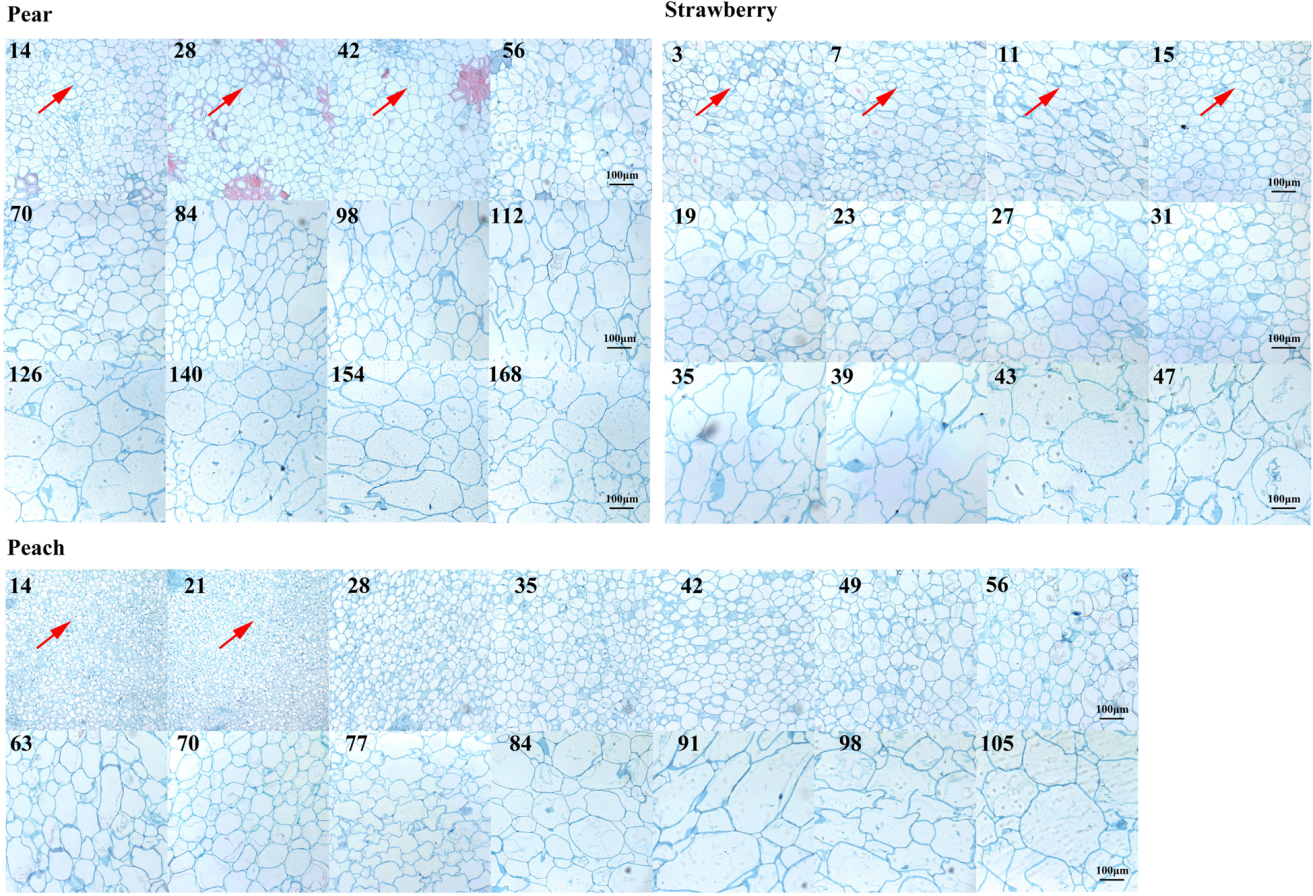
Paraffin sections of pear, peach, and strawberry fruits. Red arrows indicate the proliferation stages

To investigate the growth curves of pear, peach, and strawberry fruits, the fruit weight of each species was measured from fruitlet to maturation. Results revealed a single sigmoid pattern growth curve in pear fruits (Fig. 1a; Figure S1), but a double sigmoid pattern in peach and strawberry fruits (Fig. 1a). Further analysis of paraffin section showed that the cell number of per fruit in three fruit species mainly increased before 42, 21, and 19 days after full blooming (DAFB), respectively, and then maintained stable until to fruit ripening (Fig. 1b). In contrast, the cell size changed little before 42, 21, and 19 DAFB, respectively, and then the cell expansion was happened (Fig. 2). These results revealed that pear, peach, and strawberry fruits underwent extensive cell division before 42, 21, and 19 DAFB, respectively, then underwent cell enlargement. In addition, both the single and double sigmoid curve occurred after these days, we concluded that both single and double sigmoid patterns were a result of cell expansion, not cell division [1–4].

### Transcriptome sequencing and differential expression analyses

To uncover the difference between single and double sigmoid patterns, the fruit at fruitlet, first enlargement, interval, second enlargement, and maturation (Table S1) in peach and strawberry were selected for transcriptome sequencing. These five stage samples were designated as PP1 to PP5 in peaches (*Prunus persica*) and FA1 to FA5 in strawberries (*Fragaria × ananassa*) (Table S1), respectively. In pears (*Pyrus bretschneideri*), the fruitlet (28 DAFB), early enlargement (98 DAFB), middle enlargement (126 DAFB), late enlargement (140 DAFB), and maturation (168 DAFB) stage fruits were selected and designated as PB1 to PB5, respectively. A total of 1.41, 1.56, and 1.44 Gb raw reads were generated from pear, peach, and strawberry fruits, respectively. After removing low-quality reads, an average of 91.47, 100.01, and 92.3 Mb clean reads were used for mapping to the reference genome in pear, peach, and strawberry fruits, respectively. The average percentages of total mapped reads were 73.99, 88.64, and 72.27% in pear, peach, and strawberry fruits, respectively. A total of 31,910 genes were detected in pear fruits. Of these genes, 25,633 were commonly expressed in all tested fruits, while 1303, 231, 162, 214, and 254 were specifically expressed from PB1 to PB5, respectively (Figure S2). A total of 27,747 genes were detected in peach fruits. Of these genes, 22,480 were commonly expressed in all tested fruits, while 652, 189, 149, 113, and 291 were specifically expressed from PP1 to PP5, respectively (Figure S2). A total of 35,502 genes were detected in strawberry fruits. Of these genes, 27,023 were commonly expressed in all tested fruits, while 1156, 303, 265, 702, and 325 were specifically expressed from FA1 to FA5, respectively (Figure S2).

**Fig. 3:**
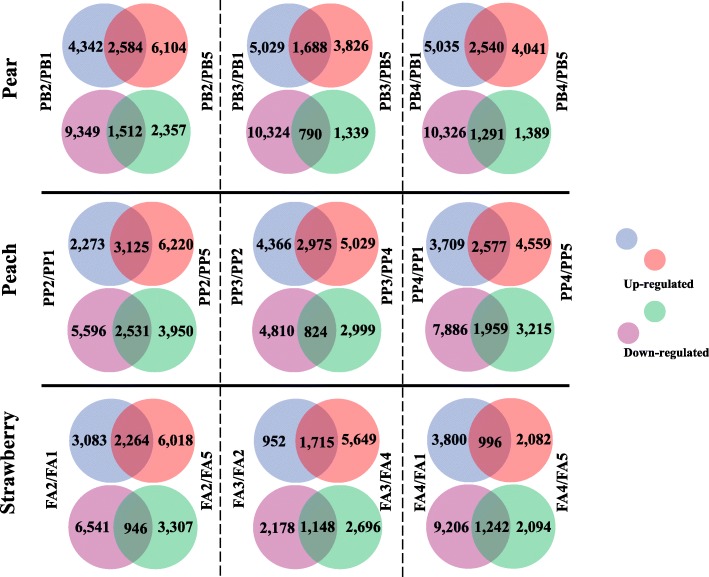
Venn diagram showing the fruit development DEGs of pear, peach, and strawberry fruits

To isolate the genes associated with fruit enlargement, a differential expression analysis was conducted to compare fruits at the rapid enlargement and fruitlet or maturation stages. In pears, 4096 (PB2-DEG), 2478 (PB3-DEG), and 3831 (PB4-DEG) genes were differentially expressed in PB2, PB3, and PB4 compared to PB1 and PB5 (Fig. 3). In peaches, 5656 (PP2-DEG) and 4536 (PP4-DEG) genes were differentially expressed in PP2 and PP4 compared to PP1 and PP5 (Fig. 3). In strawberries, 3210 (FA2-DEG) and 2238 (FA4-DEG) genes were differentially expressed in FA2 and FA4 compared to FA1 and FA5 (Fig. 3). These isolated genes were possibly associated with fruit enlargement in corresponding species. Moreover, 3799 (PP3-DEG) genes were differentially expressed between PP3 and PP2/PP4 in peaches, while 2863 (FA3-DEG) genes were differentially expressed between FA3 and FA2/FA4 (Fig. 3). The differential expression of these genes may be involved in the intervals of fruit enlargement.

### Overlap of DEGs with QTL regions related to fruit size

Fruit size, which includes fruit weight, height, width, and depth, has been widely studied in pear, peach, and strawberry fruits. In pears, fruit size was anchored by 21 QTL regions covering 1279 genes [35–37], of which, 123, 72, and 117 genes were detected in PB2-, PB3-, and PB4-DEGs, respectively (Table S2). In peaches, fruit size was anchored by 36 QTL regions covering 7100 genes [39–41], of which, 1198 and 1001 genes were detected in PP2- and PP4-DEGs, respectively (Table S2). In strawberries, fruit size was anchored by three QTL regions covering 192 genes within a 150 kb range [44, 45], of which, four genes were detected in FA2-DEG (Table S2). These results revealed that the DEGs overlapped with QTLs related to fruit size, indicating that screening for fruit enlargement candidate genes is reliable based on the transcriptome analyses of these three *Rosaceae* species.

### Conception of fruit enlargement in *Rosaceae*

Pear, peach, and strawberry fruits are *Rosaceae* species that have relatively close genetic backgrounds. However, pear fruits exhibit a single sigmoid pattern, while peach and strawberry fruits exhibit a double sigmoid pattern (Fig. 1a). These growth patterns are a result of the differences between the middle enlargement stage in pears (PB3) and interval in peaches and strawberries (PP3/FA3). In peaches, two fruit enlargement stages were mediated by PP2- and PP4-DEGs, while the interval was controlled by genes exhibiting the opposite trend in PP3-DEG compared to PP2- and PP4-DEGs. This conclusion was also observed in strawberry fruits. Therefore, it was speculated that the genes involved in the interval stages must possess correlated expression profiles with the fruit growth patterns of peaches and strawberries. In pears, the genes involved in the middle enlargement stage correlated with the expression profiles of fruit growth patterns or were independent from the genes involved in the early and late enlargement stages.

By comparing the double sigmoid pattern in peaches and strawberries, the single sigmoid pattern in pears was explained by two models. In the first model (Model I), the intervals in peach and strawberry fruits was replaced by the first and second enlargement stages, leading to the single sigmoid pattern, which was mediated by the genes associated with all three fruit enlargement stages in pears. In the second model (Model II), the intervals in peach and strawberry fruits were independent of other fruit enlargement stages, resulting in the single sigmoid pattern, which was mediated by the genes involved in the middle enlargement stage, but not the early or late enlargement stages in pears.

### Identification of cell development genes in Model I

**Fig. 4:**
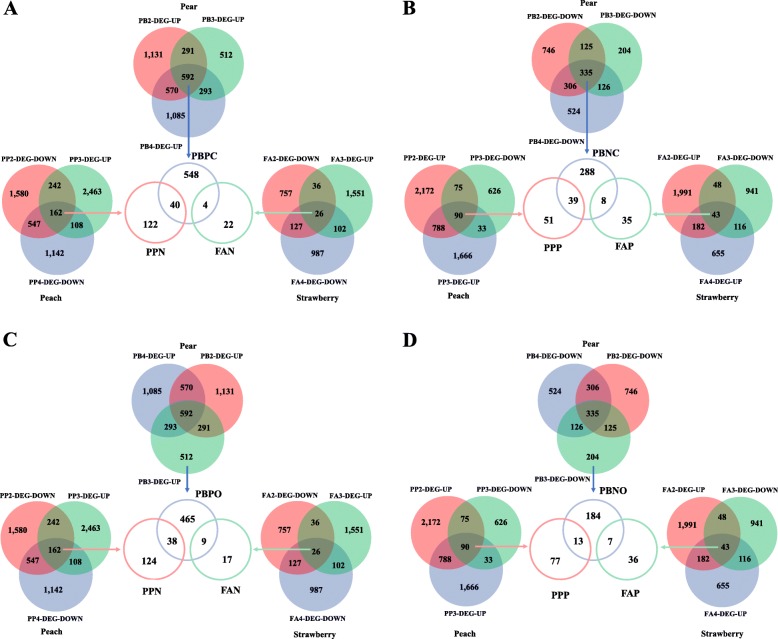
Identification of candidate genes correlated with fruit enlargement in pear, peach, and strawberry fruits in Models I and II. PB2/3/4-DEG-UP: genes upregulated in PB2, PB3, and PB4 compared to PB1 and PB5 in pears; PB2/3/4-DEG-DOWN: genes downregulated in PB2, PB3, and PB4 compared to PB1 and PB5 in pears; PP2/4-DEG-UP: genes upregulated in PP2 and PP4 compared to PP1 and PP5 in peaches; PP2/4-DEG-DOWN: genes downregulated in PP2 and PP4 compared to PP1 and PP5 in peaches; PP3-DEG-UP: genes upregulated in PP3 compared to PP2 and PP4 in peaches; PP3-DEG-DOWN: genes downregulated in PP3 compared to PP2 and PP4 in peaches; FA 2/4-DEG-UP: genes upregulated in FA2 and FA4 compared to FA1 and FA5 in strawberries; FA 2/4-DEG-DOWN: genes downregulated in FA2 and FA4 compared to FA1 and FA5 in strawberries; FA3-DEG-UP: genes upregulated in FA3 compared to FA2 and FA4 in strawberries; FA3-DEG-DOWN: genes downregulated in FA3 compared to FA2 and FA4 in strawberries; PBPC: genes upregulated in PB2-, PB3-, and PB4-DEGs; PBNC: genes downregulated in PB2-, PB3-, and PB4-DEGs; PPP: genes upregulated in PP2- and PP4-DEGs, but downregulated in PP3-DEG; PPN: genes downregulated in PP2- and PP4-DEGs, but upregulated in PP3-DEG; FAP: genes upregulated in FA2- and FA4-DEGs, but downregulated in FA3-DEG; FAN: genes downregulated in FA2- and FA4-DEGs, but upregulated in FA3-DEG

To examine the possibility of Model I, the genes that present correlated expression profiles with the two fruit growth patterns were identified in the three *Rosaceae* species. In peaches, 90 genes were upregulated in PP2- and PP4-DEGs, but downregulated in PP3-DEG; this gene set was designated as PPP. Meanwhile, 162 genes were downregulated in PP2- and PP4-DEGs, but upregulated in PP3-DEG; this gene set was designated as PPN. In strawberries, 43 genes were upregulated in FA2- and FA4-DEGs, but downregulated in FA3-DEG; this gene set was designated as FAP. Moreover, 26 genes were downregulated in FA2- and FA4-DEGs, but upregulated in FA3-DEG; this gene set was designated as FAN. In pears, 592 genes were upregulated and 335 were downregulated, which overlapped among PB2-, PB3-, and PB4-DEGs (Fig. 4a, b), correlating with the single sigmoid pattern in pears. The up- and downregulated gene sets were designated as PBPC and PBNC, respectively. Among these 927 genes, 155 were involved in cell development, including cell wall biogenesis, cell growth, and cell division (Figure S3A). However, the comparative analysis of the genes in PBPC, PPP, and FAP detected no orthologous genes among the three *Rosaceae* species, nor did the comparative analysis of the genes in PBNC, PPN, and FAN. These results were inconsistent with Model I, therefore, the genes associated with all three fruit enlargement stages in pears were different from the genes involved in the intervals in peaches and strawberries.

Although the genes in PBPC were different from the genes in PPP and FAP, it was not clear whether all the genes in PBPC were associated with fruit enlargement as the orthologous genes in peach and strawberry fruits may be included in PPN/FAN. A similar inference was made for the genes in PBNC. To verify these speculations, a comparative analysis was conducted on the genes in PBPC, PPN, and FAN. Results revealed that 44 genes in PBPC were orthologous to the genes in PPN or FAN, suggesting that these genes were not associated with the single sigmoid pattern; the remaining 548 genes in pears may be involved in fruit enlargement (Fig. 4a). Similarly, 47 genes in PBNC were orthologous to the genes in PPP or FAP, suggesting that these genes were not associated with the single sigmoid pattern; the remaining 288 genes in pears may be involved in fruit enlargement (Fig. 4b). In these 836 DEGs in pears, 90 were involved in cell development, including the cell cycle and cell wall metabolism (Table S3). Notably, of these genes, 25 were located within the fruit weight QTL regions (Table 1). Clearly, several of these genes were specifically associated with fruit enlargement in pears and the difference between the single and double sigmoid patterns may result from these genes.

**Table 1:** The identified genes from Model I and Model II overlapped with fruit size QTL markers

	Gene ID	Annotation
**Model I**	Pbr035270.1	IRK-interacting protein-like (LOC103934871)
	Pbr038258.1	uncharacterized LOC103933313 (LOC103933313)
	Pbr012946.1	transcription repressor OFP12-like (LOC103960152)
	Pbr009000.1	probable transmembrane GTPase FZO-like (LOC103957273)
	Pbr020465.2	alpha,alpha-trehalose-phosphate synthase
	Pbr033649.1	kinesin-3-like (LOC103948751)
	Pbr008977.1	chloroplast stem-loop binding protein of 41 kDa a (LOC103957266)
	Pbr008967.1	chloroplast stem-loop binding protein of 41 kDa a (LOC103957266)
	Pbr017191.1	zinc finger CCCH domain-containing protein 53-like (LOC103963791)
	Pbr039708.1	ubiquitin thioesterase OTU1-like (LOC103951354)
	Pbr022797.1	BTB/POZ domain-containing protein At1g03010 (LOC103967390)
	Pbr024215.1	rab3 GTPase-activating protein non-catalytic subunit (LOC103926877)
	Pbr030227.1	E3 SUMO-protein ligase MMS21-like (LOC103931527)
	Pbr038269.1	D-amino-acid transaminase, chloroplastic-like (LOC103932682)
	Pbr038230.1	uncharacterized LOC103932372 (LOC103932372)
	Pbr008947.1	protein MARD1-like (LOC103957220)
	Pbr022822.1	ATP-dependent zinc metalloprotease FTSH 2 (LOC103967414)
	Pbr033637.2	aminopeptidase M1-like (LOC103948738)
	Pbr039758.1	GDP-L-galactose phosphorylase 2 (LOC103951401)
	Pbr025844.1	uncharacterized LOC103927937 (LOC103927937)
	Pbr024234.1	chitin-inducible gibberellin-responsive protein 1-like (LOC103926896)
	Pbr022819.1	zuotin-like (LOC103967411)
	Pbr036709.1	uncharacterized LOC103949957 (LOC103949957)
	Pbr017194.1	uncharacterized LOC103963818 (LOC103963818)
	Pbr040573.1	chitin-inducible gibberellin-responsive protein 1-like (LOC103938203)
**Model II**	Pbr012884.1	FBD-associated F-box protein At4g10400-like (LOC103960072)
	Pbr012619.1	nudix hydrolase 18, mitochondrial-like (LOC103959904)
	Pbr003047.2	uncharacterized LOC103940404 (LOC103940404)
	Pbr020827.1	U-box domain-containing protein 13-like (LOC103944939)
	Pbr036583.1	uncharacterized LOC103935645 (LOC103935645)
	Pbr020491.1	LRR receptor-like serine/threonine-protein kinase EFR (LOC103965424)
	Pbr035276.1	F-box/LRR-repeat protein At4g14103-like (LOC103926900)
	Pbr008975.1	uncharacterized LOC103957269 (LOC103957269)
	Pbr022826.1	nuclear poly(A) polymerase 1-like (LOC103967418)
	Pbr017829.1	LRR receptor-like serine/threonine-protein kinase At3g47570
	Pbr012938.1	50S ribosomal protein L24-like (LOC103960110)
	Pbr035475.1	stress enhanced protein 2 (LOC103935051)
	Pbr014977.1	uncharacterized LOC103961245 (LOC103961245)
	Pbr017817.2	tRNA-specific 2-thiouridylase MnmA (LOC103963898)
	Pbr012885.1	---NA---
	Pbr020496.1	LRR receptor-like serine/threonine-protein kinase At3g47570 (LOC103965422)
	Pbr009970.1	C2 domain-containing protein At1g53590-like (LOC103931542)
	Pbr009987.1	probable solanesyl-diphosphate synthase 3, chloroplastic (LOC103931560)
	Pbr039779.1	65-kDa microtubule-associated protein 1-like (LOC103951420)
	Pbr038272.1	L-type lectin-domain containing receptor kinase IX.1-like (LOC103933343)
	Pbr041406.1	scarecrow-like protein 21 (LOC103938496)
	Pbr036594.1	protein DETOXIFICATION 42-like (LOC103935654)

### Identification of cell enlargement genes in Model II

To examine the possibility of Model II, the genes that mediate the middle enlargement stage, but not early or late enlargement, were isolated in pears. As a result, 512 upregulated and 204 downregulated genes were detected in PB3-DEG; these gene sets were designated as PBPO and PBNO, respectively (Fig. 4c, d). Among these 706 genes, 22 were involved in cell wall biogenesis, ubiquitin, and auxin (Figure S3B). However, comparative analysis of the genes in PBPO, PPP, and FAP detected no orthologous genes among the three *Rosaceae* species, nor did the comparative analysis of the genes in PBNO, PPN, and FAN. These results were inconsistent with Model II, therefore, the genes involved in the middle enlargement stage in pears were different from the genes involved in the intervals of peaches and strawberries.

Although the genes in PBPO were different from the genes in PPP and FAP, it was not clear whether all the genes in PBPO were associated with fruit enlargement as the orthologous genes in peach and strawberry fruits may be included in PPN/FAN. A similar inference was made for the genes in PBNO. To verify these speculations, a comparative analysis was conducted among the genes in PBPO, PPN, and FAN. Results revealed that 47 genes in PBPO were orthologous to the genes in PPN or FAN, suggesting that these genes were not associated with the single sigmoid pattern. The remaining 465 genes in pears may be involved in fruit enlargement (Fig. 4c). Similarly, 20 genes in PBNO were orthologous to the genes in PPP or FAP, suggesting that these genes were not associated with the single sigmoid pattern. The remaining 184 genes in pears may be involved in fruit enlargement (Fig. 4d). Of these 649 DEGs in pears, 63 were involved in cell development, including cell wall biogenesis, cell growth, and cell metabolism (Table S4). Notably, of these genes, 22 were located within the fruit weight QTL regions (Table 1). Clearly, several of these genes were specifically associated with fruit enlargement in pears. Thus, the difference between the single and double sigmoid patterns may be a result of Model II. Collectively, the genes detected in Models I and II in pear fruits may regulate the single sigmoid pattern.

### Expression profiles of fruit enlargement candidate genes

**Fig. 5:**
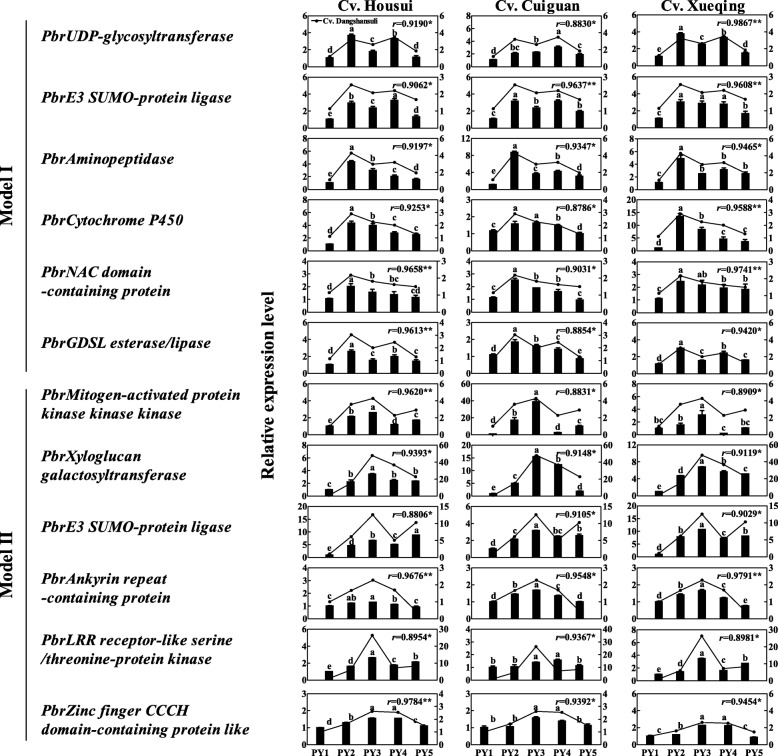
Expression profiles of the candidate genes in the ‘Housui’, ‘Cuiguan’, and ‘Xueqing’ pear cultivars. SEs were calculated from 3 biological replicates. Different lowercase letters indicate significant differences (*p* < 0.05); * and ** indicate significant correlations (*p* < 0.05 and *p* < 0.01, respectively)

To confirm the transcriptome assay, 12 candidate genes associated with cell wall, cell cycle, ubiquitination, phytohormones, cytochrome, ankyrin, transcription factor, and leucine-rich repeats (LRR) from Models I and II were selected for quantitative real-time polymerase chain reaction (qRT-PCR) analysis of pear, peach, and strawberry fruits. Results revealed that these genes had similar expression profiles as the transcriptome assay results, indicating that these findings were reliable (Figure S4). Moreover, to test whether the candidate genes exhibited similar expression profiles in different pear cultivars, the ‘Housui’, ‘Cuiguan’, and ‘Xueqing’ cultivars were investigated at the same five fruit enlargement stages and analyzed by qRT-PCR (Figure S1). Results revealed that the expression profiles of the 12 aforementioned genes in the three cultivars were almost identical to the ‘Dangshansuli’ cultivar (Fig. 5), suggesting that these candidate genes identified from ‘Dangshansuli’ were also involved in the middle enlargement stages of other pear cultivars. Therefore, these candidate genes may be associated with the single sigmoid pattern in pear fruits.

### Regulation of *bHLHs* in cell expansion-related genes

**Fig. 6:**
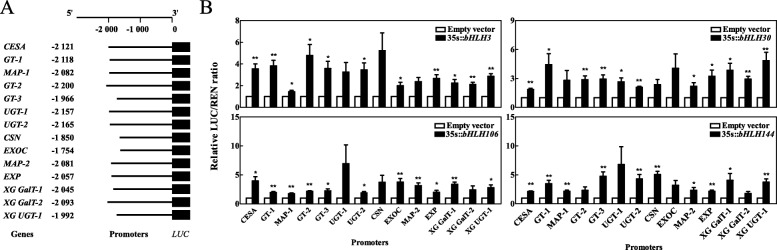
Detection of transcriptional activation of the 4 *bHLHs* in 14 candidate genes associated with the pear single sigmoid pattern. **a** Constructed effectors and reporters. **b** Dual-luciferase assay of effectors on reporters. Cell development-associated genes include *CESA* (Pbr038537.1), *GT* (Pbr016727.1, Pbr023514.1, and Pbr023516.2), *MAP* (Pbr011537.1 and Pbr039779.1), *UGT* (Pbr018679.1 and Pbr021540.1), *CSN* (Pbr025986.3), *EXOC* (Pbr029906.1), *EXP* (Pbr013129.1), *XG GalT* (Pbr036086.1 and Pbr004891.1), and *XG UGT* (Pbr005326.1). * and ** indicate significant correlations (*p* < 0.05 and *p* < 0.01, respectively)

In a previous study, *bHLH* was defined as the upstream factor regulating cell expansion-related genes in peaches [13]. In this study, four *bHLH* genes, *bHLH3* (Pbr009044.1), *bHLH30* (Pbr016145.1), *bHLH106* (Pbr013890.1), and *bHLH144* (Pbr039557.1), were detected in the genes associated with the single sigmoid pattern, as well as cell expansion-related genes, including *cellulose synthase* (*CES*), *glycosyltransferase* (*GT*), *microtubule-associated protein* (*MAP*), *UDP-glycosyltransferase* (*UGT*), *COP9 signalosome complex subunit* (*CSN*), *exocyst complex component* (*EXOC*), *expansin* (*EXP*), *xyloglucan galactosyltransferase* (*XG GalT*), and *xyloglucan glycosyltransferase* (*XG GUT*). To investigate the role of the four *bHLH* transcription factors (TFs) on the expression of cell expansion-related genes, full-length sequences of the four *bHLH* genes were amplified and inserted into pSAK277 as effectors. Additionally, ~ 2000 bp sequences in the promoter of cell expansion-related genes were inserted into pGreen 0800-LUC as reporters (Fig. 6a). The dual luciferase assay results revealed that *bHLH3*, *bHLH30*, and *bHLH106* enhanced the activities of firefly luciferase (LUC), which were driven by the promoters of 11 of the 14 selected genes, while *bHLH144* enhanced the activities of LUC and was driven by the promoters of 10 of the 14 selected genes (Fig. 6b). Notably, LUC was driven by the promoters of cellulose synthase, glycosyltransferase-1, glycosyltransferase-2, UDP-glycosyltransferase-2, expansin, xyloglucan galactosyltransferase-1, and xyloglucan glycosyltransferase, and exhibited higher activities in tobacco leaves overexpressing any of the *bHLH* genes compared to tobacco leaves transformed with an empty vector. These results suggested that the four *bHLH* TFs interacted with most promoters of cell expansion-related genes and drove their expression.

## Discussion

### Potential gene regulation network for the pear single sigmoid pattern

Single and double sigmoid patterns have been reported over the last century in *Rosaceae* fruit species [2], however, the difference between these patterns had not been explored at the molecular level until now. In this study, based on the investigated physiological phenotypes, two models of fruit enlargement in pears were proposed. By comparative transcriptome analyses of the selected fruits in three *Rosaceae* fruit species, several genes associated with the cell cycle [19–21], polysaccharides and cell development [22–24, 49–56], were detected in Models I and II (Tables S3 and S4). Therefore, models I and II play important roles in generating the single sigmoid pattern.

In addition to the above genes, several transcription factors were also detected in Models I and II (Table 1; Tables S3 and S4), including *zinc finger proteins* (*ZFPs*), which control cell size during plant organogenesis [57], and *bHLHs*, which regulate cell extension by transducing auxin signaling [27]. *bZIP* mediates cell expansion by affecting the biosynthesis and signal transduction of gibberellins and auxins [24, 58], while ankyrin repeat proteins enhance auxin biosynthesis via *bZIP* [59]. *WRKY* and *NAC* are involved in cell development [30, 60, 61], as well as auxin biosynthesis [62, 63], while *proliferating cell factor* (*TCP*) and *MYB* are only associated with auxin biosynthesis [64, 65]. Clearly, auxin signal transduction may be involved in the middle enlargement stage of pear fruits.

**Fig. 7:**
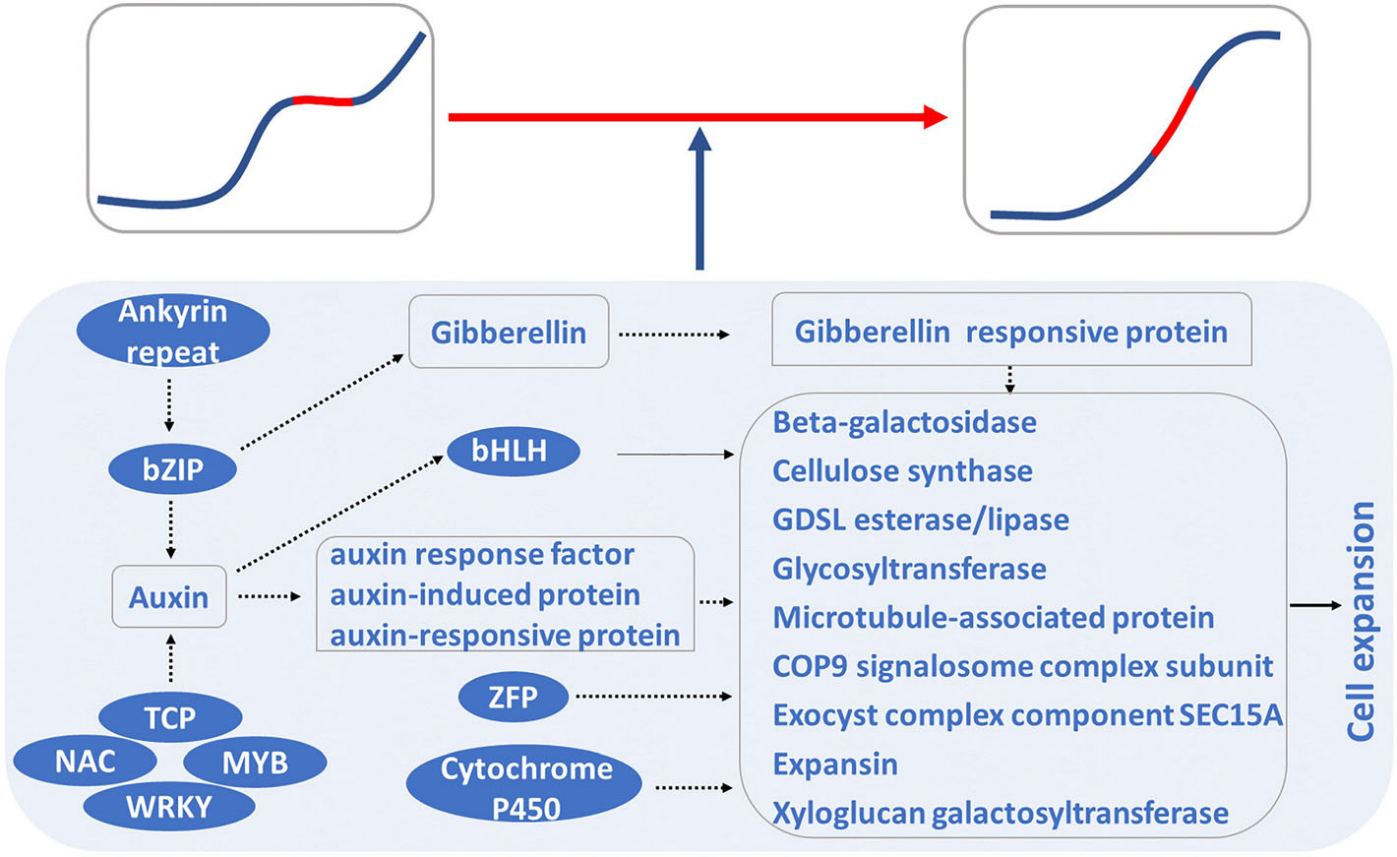
Potential gene regulation network of the pear single sigmoid pattern showing *TCP*, *NAC*, *MYB*, *WRKY*, and ankyrin-mediated *bZIP*-regulated auxin expression. *bZIP* triggered gibberellin signal transduction to activate gibberellin-responsive proteins. Auxin-induced factors along with *bHLH*, *ZFP*, cytochrome P450, and gibberellin-responsive proteins promoted the expression of genes related to cell expansion, which resulted in the middle enlargement of pear fruits

In this study, auxin-induced factors, including auxin response factor, auxin-induced proteins, and auxin-responsive proteins, were detected in Models I and II, which are associated with cell expansion [66, 67]. Moreover, the detected cytochrome p450 proteins stimulate plant organ growth by increasing cell size [17]. The transcriptional activation of the four *bHLHs* in 14 candidate genes associated with the pear single sigmoid pattern of the two models was also confirmed by the dual-luciferase assay. These results revealed that the promoters of the 14 genes were activated by *bHLHs* (Fig. 6). Based on these results, a potential gene regulation network was proposed for the pear single sigmoid pattern (Fig. 7). This network indicates that phytohormone auxin was regulated by *MYB*, *WRKY*, *TCP*, *NAC*, and ankyrin-mediated *bZIP*, which thereby triggered the expression of *bHLH* and auxin-induced factors. Simultaneously, *bZIP* triggered gibberellin signal transduction to activate gibberellin-responsive proteins. Auxin-induced factors along with *bHLH*, *ZFP*, *cytochrome P450*, and *gibberellin-responsive proteins* promoted the expression of *beta-galactosidase*, *cellulose synthase*, *GDSL esterase*/*lipase*, *Glycosyltransferase*, *microtubule-associated protein*, *COP9 signalosome complex subunit*, *exocyst complex component SEC15A*, *expansin*, and *xyloglucan galactosyltransferase* to promote cell expansion, which resulted in the middle enlargement of pear fruits.

### Positive selection was observed in pear and peach fruit enlargement genes

Plant domestication is a long-term, people-based plant interaction that favors the alleles of genes that control certain traits of interest in increased frequency [68]. In fleshy fruits, fruit enlargement is a typical phenotype observed during fruit development and maturation. In a previous study, tomato fruits exhibited a single sigmoid pattern during fruit growth [2], and fruit mass was domesticated from the small fruits of their ancestors to the big fruits of modern tomatoes [55, 69]. The similar domestication of fruit size was detected in pears [70], peaches [71, 72], apples [73] and sweet cherries [43].

In this study, the dynamic weight of developing and mature pear, peach, and strawberry fruits was measured. Results confirmed that pear fruits exhibited a single sigmoid pattern during fruit growth, while peach and strawberry fruits exhibited a double sigmoid pattern. However, it is unclear whether the fruit growth patterns of these three *Rosaceae* species undergo positive selection. Previously, the selective sweep regions of fruit size were isolated in peach and pear fruits [70, 72, 74]. In peaches, 707 and 507 genes correlated with the first and second enlargement stages, respectively, and were located within the selective sweep regions. Moreover, 395 genes correlated with the first and second enlargement stages [71, 72]. These results indicate that the first and second enlargement stages were domesticated during the evolution of these species (Table S5). Similarly, in pears, 66, 32, and 74 genes correlated with the early, middle, and late enlargement stages, respectively, and were located within the selective sweep regions. Moreover, 21 genes correlated with the three fruit enlargement stages (Table S5). These results suggest that the three fruit enlargement stages were domesticated during the evolution of these species. Collectively, these findings indicate that each fruit enlargement stage was domesticated in pear and peach fruits.

In previous studies, the double sigmoid pattern of peach fruits was explained by two hypotheses: the competition for assimilates between the seed and pericarp, and the hormonal control of pericarp growth by the seed [6]. In this study, these hypotheses may co-exist in the three fleshy fruits. First, strawberry fruit development is dependent on seed fertilization-induced hormones [75], indicating that hormones are necessary for fruit enlargement. Theoretically, hormone-controlled fruit enlargement should not be interrupted. However, when strawberry fruits were bleaker in color (Figure S5), both hormones and assimilates were used for anthocyanin biosynthesis or fruit enlargement, leading to slow fruit growth. Similarly, when peach fruits exhibited stone hardening (Figure S5), both hormones and assimilates were provided for endocarp lignification or fruit enlargement, inducing slow fruit growth. In contrast, due to the lack of dramatic changes observed during pear fruit development (Fig. 1a), both hormones and assimilates were used for fruit enlargement, which resulted in the single sigmoid pattern.

## Conclusions

In this study, pear fruits exhibited a single sigmoid pattern, while peach and strawberry fruits exhibited a double sigmoid pattern. These patterns resulted from cell expansion, not cell division. By comparative transcriptome analysis among pear, peach, and strawberry fruits, 836 genes were found to be associated with all three fruit enlargement stages in pear fruits (Model I), while 649 genes were associated with middle enlargement stage, not early or late enlargement (Model II). Therefore, the pear single sigmoid pattern appeared to be coordinately mediated by these proposed models. Interestingly, most genes in these models were annotated and correlated with cell development. Moreover, 47 genes were located within the QTL regions related to fruit weight. Based on previous reports and the dual-luciferase assay, a potential gene network of the single sigmoid pattern was drafted in this study (Fig. 7).

## Methods

### Plant materials

The ‘Dangshansuli’ (*P. bretschneideri* Rehd.), ‘Cuiguan’ (*P. pyrifolia Nakai*), ‘Xueqing’ (*P. bretschneideri* Rehd.), and’ Housui’ (*P. serotina* Rhed.) pear cultivars were maintained at the Jiangpu orchard of Nanjing Agricultural University, Nanjing, China. Pear fruits were collected every 14 days from 14 DAFB to maturation. The late-maturing ‘Liangyuan’ peach cultivar (*P. persica*) was maintained at the inner Zhen Jiang Agricultural Academy, Hangzhou, China. Peach fruits were collected every 7 days from 14 DAFB to maturation. The ‘Hongyan’ strawberry cultivar (*F. × ananassa*) was grown in a greenhouse at Zhen Jiang Agricultural Academy, China. A total of 300 flowers were selected and marked at the initial flowering stage; the fruits derived from these flowers were collected every 4 days from three DAFB to maturation. Fruit weight was measured using 15 fruits from each period for all fruit species.

‘Danshansuli’, ‘Liangyuan’, and ‘Hongyan’ fruits were cut into pieces and fixed in a fixative solution (90 mL 50% ethanol, 5 mL formol, 5 mL glacial acetic acid) for > 24 h. After dehydration by gradient alcohol application and embedding into paraffin, samples were sliced using an RM2016 LEICA slicer (Leica, Wetzlar, German). Pictures of the paraffin sections were visualized using a Nikon ECLIPSE E100 system (10 × 10) (Nikon, Tokyo, Japan). Cell numbers were recorded using imageJ 1.47v (National Institute of Health, Bethesda, USA).

Moreover, to analyze the expression profiles of whole predicted genes during fruit swelling in pear, peach, and strawberry fruits, each collection had a minimum of three independent replicates. The collected pear and peach fruits were snap frozen in liquid nitrogen and ground into a fine powder. Similarly, after removing the seeds from the surface of strawberry fruits, the remaining tissues were mashed in liquid nitrogen and stored at − 80 °C for further analysis.

### Library preparation and sequencing

Total RNA was extracted using an RNAprep Pure Plant kit (Tiangen Biotech, Beijing, China). RNA degradation and contamination were monitored on 1% agarose gel. RNA purity was checked using a NanoPhotometer® spectrophotometer (Implen, CA, USA). RNA concentrations were measured using a Qubit® RNA Assay kit in a Qubit® 2.0 flurometer (Life Technologies, CA, USA). RNA integrity was assessed using an RNA Nano 6000 Assay kit on a Bioanalyzer 2100 system (Agilent Technologies, CA, USA). The RNA integrity number (RIN) of pear, peach, and strawberry fruits was > 8.8, which met the requirement for building libraries. After qualitative and quantitative detections of total RNA, ribosomal RNA (rRNA) was depleted using an Epicentre Ribo-zero™ rRNA Removal kit (Epicentre, WI, USA). rRNA-deleted RNA fragmentation was used to synthesize first and second cDNA strands with a random hexamer primer in NEBNext First Strand Synthesis Reaction buffer (5×). Then, the NEBNext adaptor was added to adenylated DNA fragments followed by purification and screening of cDNA fragments, which were 150–200 bp in length. A truSeq PP Cluster kit v3-cBot-HS (Illumina, CA, USA) was used to perform clustering. Then, the libraries for ‘Danshansuli’, ‘Liangyuan’, and ‘Hongyan’ were sequenced on an Illumina Hiseq 4000 platform (Illumina, CA, USA) with three replicates.

### Quality control and transcriptome assembly

Quality control of raw data (raw reads) in fastq format was conducted according to previously described methods [76]. After removing unqualified raw reads, clean reads were mapped to the reference genomes using Bowtie2 v2.2.8 and HISAT2 v2.0.4 [77]. The reference genomes of pear, peach, and strawberry fruits consisted of the *Pyrus* genome v1.1 (http://peargenome.njau.edu.cn), *Prunus*_*persica*_v2.0.a1 (https://www.ncbi.nlm.nih.gov) and *Fragaria_vesca*_v2.0.a.1 (http://ftp.bioinfo.wsu.edu), respectively. The clean reads mapped to the reference genomes and assembled using StringTie v1.3.1 following a reference-based approach [78]. StringTie was used for its novel network flow algorithm and optional de novo assembly step for assembling and quantifying full-length transcripts that represent multiple splice variants for each gene locus.

### Differential expression analysis

Gene expression levels were calculated based on the fragments per kilobase of exon per million fragments mapped (FPKM) reads using Cuffdiff v2.1.1 [79]. After filtering unreliable data in python scripts (https://github.com/Peims/Batch-screening-unqualified-data). A differential expression analysis was conducted using limma packages (https://bioconductor.org/packages/limma/). The criterion for distinguishing significant gene differences was *p* < 0.05 [80, 81].

### Gene function annotation

Gene Ontology (GO) enrichment analysis of the DEGs was implemented using PlantRegMap (http://plantregmap.cbi.pku.edu.cn/). The screening criteria of significantly enriched terms was *p* < 0.05. KOBAS v3.0 (http://kobas.cbi.pku.edu.cn/) software was used to calculate the enrichment of KEGG pathway DEGs. Gene functional annotations were conducted using local BLAST 2.2.29+ against the NCBI NR database.

### Chromosome location of DEGs and orthologous analysis

The location of DEGs was retrieved based on mRNA information. DEGs were located within the reported QTL regions were statistically analyzed in python script (https://github.com/Peims/Calculate-the-distance-between-the-gene-and-the-marker). An orthologous genes analysis was conducted using OrthoFinder v2.3.3 [82].

### qRT-PCR

Total RNA was extracted using a Plant Total RNA Isolation kit plus (Gore Gene, Chengdu, China). The first strand of cDNA was synthesized using FastKing gDNA Dispelling RT SuperMix kits (Tiangen Biotech, Beijing, China), which was subsequently used as a template for qRT-PCR. qRT-PCR was performed on a Lightcycle-480 system using LightCycler 480 SYBR Green I Master mix (Roche, Basel, Sweden). The reaction mixture was conducted as previously reported [74]. The gene-specific primers used in this study are provided (Table S6). All PCR experiments were performed using three independent biological samples, which included three technical replicates. The pear *SNF*, peach *TEF*, and strawberry *DBP* genes were used as normalizers. An independent sample t-test was used to analyze the qRT-PCR results between each sample. A correlation analysis between transcriptome and qRT-PCR results was conducted in python script (https://github.com/Peims/calculate-the-pearsoner).

### Vector constructs and dual luciferase assays

To detect the transcriptional activation of four *bHLHs* on 14 candidate genes associated with the single sigmoid pattern of Models I and II, full-length sequences of the four *bHLHs* were amplified using specific primers (Table S7). Phanta® Max Super-Fidelity DNA polymerase (P505, Vazyme Biotech Co., Ltd., China) was introduced into the pSAK277 vector to construct the effectors (Fig. 6a). The ~ 2000 bp promoter sequence of the 14 genes was inserted into the pGreen 0800-LUC vector to construct reporters. Then, the correctly recombinant plasmids were transformed into the *Agrobacterium* strain, GV3101, using pSoup for pGreen 0800-LUC vector transformation. The GV3101 strain contained an effector and reporter was co-infiltrated into tobacco leaves [83]. The empty pSAK277 transient expression vector was used as the negative control. For each promoter-TF comparison, at least six infiltrated leaves using the same *Agrobacterium* culture were used in the dual-luciferase assay. LUC and Renillia luciferase (REN) activity were assayed using a Dual-Luciferase® Reporter Assay system (Promega, WI, USA) following the manufacturer’s instructions. Relative LUC activities were calculated as the ratio of LUC/REN for each comparison and normalized to the control in each experiment. Statistical analyses were conducted using SPSS v17.0 software (SPSS Inc., CHI, USA). Student’s *t*-tests were performed to determine significant differences (*p* < 0.05).

## Supplementary information

**Additional file 1: Figure S1.** Measurements of fruit weight in Cvs. ‘Housui’, ‘Cuiguan’ and ‘Xueqing’.

**Additional file 2: Figure S2.** Commonly and specially expressed genes in five period of three fruit species.

**Additional file 3: Figure S3.** GO enrichment of candidate genes identified in Model I and Model II.

**Additional file 4: Figure S4.** Expression profiles of the differential expressed genes related to fruit enlargements in pears, peaches and strawberries, respectively.

**Additional file 5: Figure S5.** The pictures of strawberries and peaches in the intervals.

**Additional file 6: Table S1.** Stages of peaches and strawberries selected for transcriptome sequencing.

**Additional file 7: Table S2.** Overlap of differential expressed genes with QTL regions related to fruit size.

**Additional file 8: Table S3.** Candidate genes involved into fruit enlargement in Model I.

**Additional file 9: Table S4.** Candidate genes involved into fruit enlargement in Model II.

**Additional file 10: Table S5.** The gene involved into fruit enlargements undergo positive selection in both pears and peaches.

**Additional file 11: Table S6.** Primers for real-time quantitative PCR.

**Additional file 12: Table S7.** Primers for dual luciferase assay.

## Data Availability

Illumina sequencing data from ‘pears, peaches and strawberries’ were deposited in NCBI SRA database under accession number SRP238133, bioProject accession: PRJNA596556. Data generated or analyzed during this study are included in this article and its supplementary information files.

## Abbreviations

DAFB: Days after full blooming
DEG: Differentially expressed gene
FA: Strawberry
FAN: Genes downregulated in FA2- and FA4-DEGs, but upregulated in FA3-DEG in strawberries
FAP: Genes upregulated in FA2- and FA4-DEGs, but downregulated in FA3-DEG in strawberries
PB: Pear
PBPC (PBNC): Overlapping downregulated and upregulated genes among PB2-, PB3-, and PB4-DEGs in pears
PP: Peach
PPN: Genes downregulated in PP2- and PP4-DEGs, but upregulated in PP3-DEG in peaches
PPP: Genes upregulated in PP2- and PP4-DEGs, but downregulated in PP3-DEG in peaches
qRT-PCR: Quantitative real-time polymerase chain reaction
QTL: Quantitative trait locus

## References

[1] Cuevas J, Salvador-Sola FJ, Gavilán J, Lorente N, Hueso JJ, González-Padierna CM (2003). Loquat fruit sink strength and growth pattern. Sci Hortic (Amsterdam).

[2] Dennis Jr, FG. Fruit development. In: Tesar MB, editor. Physiological Basis of Crop Growth and Development. Madison: American Society of Agronomy; 1988. p. 273.

[3] Farinati S, Rasori A, Varotto S, Bonghi C (2017). Rosaceae fruit development, ripening and post-harvest: an epigenetic perspective. Front Plant Sci.

[4] Opara LU (2000). Fruit growth measurement and analysis. Hortic Rev.

[5] Rasori A, Ziliotto F, Botton A, Tadiello A, Trainotti L, Bonghi C, Ramina A (2010). Hormonal cross talk between fruit and seed throughout development and maturation in peach. Acta Hortic.

[6] Pavel EW, DeJong TM (1993). Relative growth rate and its relationship to compositional changes of nonstructural carbohydrates in the mesocarp of developing peach fruits. J Am Soc Hortic Sci.

[7] Miura H, Imada S, Yabuuchi S (1990). Double sigmoid growth curve of strawberry fruit. J JPN Soc Hortic Sci.

[8] Kassai T, Mosoni P, Patyi R, Dénes F (2002). Investigation of the dynamics of fruit growth in two strawberry varieties. Acta Hortic.

[9] Ledesma NA, Nakata M, Sugiyama N (2008). Effect of high temperature stress on the reproductive growth of strawberry cvs. ‘Nyoho’ and ‘Toyonoka’. Sci Hortic.

[10] Vallarino JG, e Lima FD, Soria C, Tong H, Pott DM, Willmitzer L, Fernie AR, Nikoloski Z, Osorio S (2018). Genetic diversity of strawberry germplasm using metabolomic biomarkers. Sci Rep.

[11] Kim SK, Bae RN, Hwang H, Kim MJ, Sung HR, Chun C (2010). Comparison of bioactive compounds contents in different fruit tissues of june-bearing strawberry cultivars. J Am Soc Hortic Sci.

[12] Lee CH, Min JH, Kim TI, Kim JG, Matsumoto K, Kim DY, Hwang YS (2011). Comparison of wall polymers among three genetically closely related strawberry cultivars with different fruit firmness. Hortic Environ Biotechnol.

[13] Gu C, Zhou Y, Shu W, Cheng H, Wang L, Han Y, Zhang Y, Yu M, Joldersma D, Zhang S (2018). RNA-Seq analysis unveils gene regulation of fruit size cooperatively determined by velocity and duration of fruit swelling in peach. Physiol Plant.

[14] Mizukami Y (2001). A matter of size: developmental control of organ size in plants. Curr Opin Plant Biol.

[15] Sugimoto-Shirasu K, Roberts K (2003). ‘Big it up’: endoreduplication and cell-size control in plants. Curr Opin Plant Biol.

[16] Harada T, Kurahashi W, Yanai M, Wakasa Y, Satoh T (2005). Involvement of cell proliferation and cell enlargement in increasing the fruit size of *Malus* species. Sci Hortic (Amsterdam).

[17] Anastasiou E, Kenz S, Gerstung M, MacLean D, Timmer J, Fleck C, Lenhard M (2007). Control of plant organ size by KLUH/CYP78A5-dependent intercellular signaling. Dev Cell.

[18] Disch S, Anastasiou E, Sharma VK, Laux T, Fletcher JC, Lenhard M (2006). The E3 ubiquitin ligase BIG BROTHER controls *Arabidopsis* organ size in a dosage-dependent manner. Curr Biol.

[19] Dewitte W, Scofield S, Alcasabas AA, Maughan SC, Menges M, Braun N (2007). *Arabidopsis* CYCD3 D-type cyclins link cell proliferation and endocycles and are rate-limiting for cytokinin responses. Proc Nat Acad Sci U S A.

[20] Ribone PA, Capella M, Chan RL (2015). Functional characterization of the homeodomain leucine zipper I transcription factor *AtHB13* reveals a crucial role in *Arabidopsis* development. J Exp Bot.

[21] Smékalová V, Luptovčiak I, Komis G, Šamajová O, Ovečka M, Doskočilová A (2014). Involvement of YODA and mitogen activated protein kinase 6 in *Arabidopsis* post-embryogenic root development through auxin up-regulation and cell division plane orientation. New Phytol.

[22] Hu H, Zhang R, Tao Z, Li X, Li Y, Huang J, Li X, Han X, Feng S, Zhang G, Peng L (2018). Cellulose synthase mutants distinctively affect cell growth and cell wall integrity for plant biomass production in *Arabidopsis*. Plant Cell Physiol.

[23] Pesquet E, Korolev AV, Calder G, Lloyd CW (2010). The microtubule-associated protein AtMAP70-5 regulates secondary wall patterning in *Arabidopsis* wood cells. Curr Biol.

[24] Roach MJ, Mokshina NY, Badhan A, Snegireva AV, Hobson N, Deyholos MK, Gorshkova TA (2011). Development of cellulosic secondary walls in flax fibers requires beta-galactosidase. Plant Physiol.

[25] Scheible WR, Pauly M (2004). Glycosyltransferases and cell wall biosynthesis: novel players and insights. Curr Opin Plant Biol.

[26] Fukazawa J, Sakai T, Ishida S, Yamaguchi I, Kamiya Y, Takahashi Y (2000). Repression of shoot growth, a *bZIP* transcriptional activator, regulates cell elongation by controlling the level of gibberellins. Plant Cell.

[27] Zhou H, Lin-Wang K, Wang H, Gu C, Dare AP, Espley RV, He H, Allan AC, Han Y (2015). Molecular genetics of blood-fleshed peach reveals activation of anthocyanin biosynthesis by *NAC* transcription factors. Plant J.

[28] Yao G, Ming M, Allan AC, Gu C, Li L, Wu X, Wang R, Chang Y, Qi K, Zhang S, Wu J (2017). Map-based cloning of the pear gene *MYB114* identifies an interaction with other transcription factors to coordinately regulate fruit anthocyanin biosynthesis. Plant J.

[29] Yi K, Menand B, Bell E, Dolan L (2010). A basic helix-loop-helix transcription factor controls cell growth and size in root hairs. Nat Genet.

[30] Yang L, Zhao X, Yang F, Fan D, Jiang Y, Luo K (2016). *PtrWRKY19*, a novel *WRKY* transcription factor, contributes to the regulation of pith secondary wall formation in *Populus trichocarpa*. Sci Rep.

[31] Gu C, Wang L, Wang W, Zhou H, Ma B, Zheng H, Fang T, Ogutu C, Vimolmangkang S, Han Y (2016). Copy number variation of a gene cluster encoding endopolygalacturonase mediates flesh texture and stone adhesion in peach. J Exp Bot.

[32] Illa-Berenguer E, Van Houten J, Huang Z, van der Knaap E (2015). Rapid and reliable identification of tomato fruit weight and locule number loci by QTL-seq. Theor Appl Genet.

[33] Chakrabarti M, Zhang N, Sauvage C, Muños S, Blanca J, Cañizares J (2013). A cytochrome P450 regulates a domestication trait in cultivated tomato. Proc Natl Acad Sci U S A.

[34] Grandillo S, Ku H, Tanksley S (1999). Identifying the loci responsible for natural variation in fruit size and shape in tomato. Theor Appl Genet.

[35] Wu J, Li LT, Li M, Khan MA, Li X, Chen H, Yin H, Zhang S (2014). High-density genetic linkage map construction and identification of fruit-related QTLs in pear using SNP and SSR markers. J Exp Bot.

[36] Yamamoto T, Terakami S, Takada N, Nishio S, Onoue N, Nishitani C (2014). Identification of QTLs controlling harvest time and fruit skin color in Japanese pear (*Pyrus pyrifolia* Nakai). Breed Sci.

[37] Zhang R, Wu J, Li X, Khan MA, Chen H, Korban SS, Zhang S (2013). An AFLP, SRAP, and SSR genetic linkage map and identification of QTLs for fruit traits in pear (*Pyrus* L.). Plant Mol Biol Rep.

[38] Kenis K, Keulemans J, Davey MW (2008). Identification and stability of QTLs for fruit quality traits in apple. Tree Genet Genomes.

[39] Zeballos JL, Abidi W, Giménez R, Monforte AJ, Moreno MA, Gogorcena Y (2016). Mapping QTLs associated with fruit quality traits in peach [*Prunus persica* (L.) Batsch] using SNP maps. Tree Genet Genomes.

[40] Da Silva LC, Bassi D, Bianco L, Pacheco I, Pirona R, Rossini L (2015). Genetic dissection of fruit weight and size in an F2 peach (*Prunus persica* (L.) Batsch) progeny. Mol Breeding.

[41] Mora JRH, Micheletti D, Bink M, Van de Weg E, Cantin C (2017). Integrated QTL detection for key breeding traits in multiple peach progenies. BMC Genomics.

[42] Rosyara UR, Bink MCAM, van de Weg E, Zhang G, Wang D, Sebolt A (2013). Fruit size QTL identification and the prediction of parental QTL genotypes and breeding values in multiple pedigreed populations of sweet cherry. Mol Breeding.

[43] Zhang G, Sebolt AM, Sooriyapathirana SS, Wang D, Bink MC, Olmstead JW (2010). Fruit size QTL analysis of an F1 population derived from a cross between a domesticated sweet cherry cultivar and a wild forest sweet cherry. Tree Genet Genomes.

[44] Hancock JF, Sooriyapathirana SS, Bassil NV, Stegmeir T, Cai L, Finn CE, Van de Weg E, Weebadde CK (2016). Public availability of a genotyped segregating population may foster marker assisted breeding (MAB) and quantitative trait loci (QTL) discovery: an example using strawberry. Front Plant Sci.

[45] Verma S, Zurn JD, Salinas N, Mathey MM, Denoyes B, Hancock JF, Finn CE, Bassil NV, Whitaker VM (2017). Clarifying sub-genomic positions of QTLs for flowering habit and fruit quality in U.S. strawberry (*Fragaria* × *ananassa*) breeding populations using pedigree-based QTL analysis. Hortic Res.

[46] Wu J, Wang Z, Shi Z, Zhang S, Ming R, Zhu S (2013). The genome of the pear (*Pyrus bretschneideri* Rehd.). Genome Res.

[47] Verde I, Abbott AG, Scalabrin S, Jung S, Shu S, Marroni F (2013). The high-quality draft genome of peach (*Prunus persica*) identifies unique patterns of genetic diversity, domestication and genome evolution. Nat Genet.

[48] Shulaev V, Sargent DJ, Crowhurst RN, Mockler TC, Folkerts O, Delcher AL (2011). The genome of woodland strawberry (*Fragaria vesca*). Nat Genet.

[49] Madson M, Dunand C, Li X, Verma R, Vanzin GF, Caplan J, Shoue DA, Carpita NC, Reiter WD (2003). The *MUR3* gene of *Arabidopsis* encodes a xyloglucan galactosyltransferase that is evolutionarily related to animal exostosins. Plant Cell.

[50] Chebli Y, Geitmann A (2017). Cellular growth in plants requires regulation of cell wall biochemistry. Curr Opin Cell Biol.

[51] Gibeaut DM (2000). Nucleotide sugars and glycosyltransferases for synthesis of cell wall matrix polysaccharides. Plant Physiol Bioch.

[52] Nahlik K, Dumkow M, Bayram O, Helmstaedt K, Busch S, Valerius O (2010). The COP9 signalosome mediates transcriptional and metabolic response to hormones, oxidative stress protection and cell wall rearrangement during fungal development. Mol Microbiol.

[53] Zhang B, Zhang L, Li F, Zhang D, Liu X, Wang H, Xu Z, Chu C, Zhou Y (2017). Control of secondary cell wall patterning involves xylan deacetylation by a GDSL esterase. Nat Plants.

[54] Rajangam AS, Kumar M, Aspeborg H, Guerriero G, Arvestad L, Pansri P (2008). MAP 20, a microtubule-associated protein in the secondary cell walls of hybrid aspen, is a target of the cellulose synthesis inhibitor 2,6-dichlorobenzonitrile. Plant Physiol.

[55] Lin T, Zhu G, Zhang J, Xu X, Yu Q, Zheng Z (2014). Genomic analyses provide insights into the history of tomato breeding. Nat Genet.

[56] Cole RA, Synek L, Zarsky V, Fowler JE (2005). SEC8, a subunit of the putative *Arabidopsis* exocyst complex, facilitates pollen germination and competitive pollen tube growth. Plant Physiol.

[57] Schiessl K, Kausika S, Southam P, Bush M, Sablowski R (2012). JAGGED controls growth anisotropy and cooridination between cell size and cell cycle during plant organogenesis. Curr Biol.

[58] Weiste C, Dröge-Laser W (2014). The *Arabidopsis* transcription factor *bZIP11* activates auxin-mediated transcription by recruiting the histone acetylation machinery. Nat Commun.

[59] Böttner S, Iven T, Carsjens CS, Dröge-Laser W (2009). Nuclear accumulation of the ankyrin repeat protein ANK1 enhances the auxin-mediated transcription accomplished by the *bZIP* transcription factors *BZI-1* and *BZI-2*. Plant J.

[60] Hussey SG, Mizrachi E, Spokevicius AV, Bossinger G, Berger DK, Myburg AA (2011). *SND2*, a *NAC* transcription factor gene, regulates genes involved in secondary cell wall development in *Arabidopsis* fibres and increases fibre cell area in Eucalyptus. BMC Plant Biol.

[61] Yamaguchi M, Demura T (2010). Transcriptional regulation of secondary wall formation controlled by NAC domain proteins. Plant Biotech.

[62] Ding Z, Yan J, Li C, Li G, Wu Y, Zheng S (2015). Transcription factor *WRKY46* modulates the development of *Arabidopsis* lateral roots in osmotic/salt stress conditions via regulation of ABA signaling and auxin homeostasis. Plant J.

[63] Huh SU, Lee SB, Kim HH, Paek KH (2012). *ATAF2*, a *NAC* transcription factor, binds to the promoter and regulates *NIT2* gene expression involved in auxin biosynthesis. Mol Cells.

[64] Koyama T, Mitsuda N, Seki M, Shinozaki K, Ohme-Takagi M (2010). *TCP* transcription factors regulate the activities of ASYMMETRIC LEAVES1 and miR164, as well as the auxin response, during differentiation of leaves in *Arabidopsis*. Plant Cell.

[65] Kwon Y, Kim JH, Nguyen HN, Jikumaru Y, Kamiya Y, Hong SW, Lee H (2013). A novel *Arabidopsis MYB-like* transcription factor, MYBH, regulates hypocotyl elongation by enhancing auxin accumulation. J Exp Bot.

[66] Schruff MC, Spielman M, Tiwari S, Adams S, Fenby N, Scott RJ (2006). The *AUXIN RESPONSE FACTOR 2* gene of *Arabidopsis* links auxin signalling, cell division, and the size of seeds and other organs. Development.

[67] Spartz AK, Lee SH, Wenger JP, Gonzalez N, Itoh H, Inzé D, Peer WA, Murphy AS, Overvoorde PJ, Gray WM (2012). The *SAUR19* subfamily of *SMALL AUXIN UP RNA* genes promote cell expansion. Plant J.

[68] Wang L, Stec A, Hey J, Lukens L, Doebley J (1999). The limits of selection during maize domestication. Nature.

[69] Zhu G, Wang S, Huang Z, Zhang S, Liao Q, Zhang C (2018). Rewiring of the fruit metabolome in tomato breeding. Cell.

[70] Wu J, Wang Y, Xu J, Korban SS, Fei Z, Tao S (2018). Diversification and independent domestication of Asian and European pears. Genome Biol.

[71] Cao K, Zheng Z, Wang L, Liu X, Zhu G, Fang W (2014). Comparative population genomics reveals the domestication history of the peach, *Prunus persica*, and human influences on perennial fruit crops. Genome Biol.

[72] Yu Y, Fu J, Xu Y, Zhang J, Ren F, Zhao H (2018). Genome re-sequencing reveals the evolutionary history of peach fruit edibility. Nat Commun.

[73] Yao J, Xu J, Cornille A, Tomes S, Karunairetnam S, Luo Z (2015). A microRNA allele that emerged prior to apple domestication may underlie fruit size evolution. Plant J.

[74] Cao K, Zhou Z, Wang Q, Guo J, Zhao P, Zhu G, Fang W, Chen C, Wang X, Wang X, Tian Z, Wang L (2016). Genome-wide association study of 12 agronomic traits in peach. Nat Commun.

[75] Kang C, Darwish O, Geretz A, Shahan R, Alkharouf N, Liu Z (2013). Genome-scale transcriptomic insights into early-stage fruit development in woodland strawberry *Fragaria vesca*. Plant Cell.

[76] Pei M, Gu C, Zhang S (2019). Genome-wide identification and expression analysis of genes associated with peach (*Prunus persica*) fruit ripening. Sci Hortic (Amsterdam).

[77] Langmead B, Salzberg SL (2012). Fast gapped-read alignment with Bowtie 2. Nat Methods.

[78] Pertea M, Kim D, Pertea GM, Leek JT, Salzberg SL (2016). Transcript-level expression analysis of RNA-seq experiments with HISAT, StringTie and Ballgown. Nat Protoc.

[79] Trapnell C, Williams BA, Pertea G, Mortazavi A, Kwan G, Van Baren MJ, Salzberg SL, Wold BJ, Pachter L (2010). Transcript assembly and quantification by RNA-seq reveals unannotated transcripts and isoform switching during cell differentiation. Nat Biotechnol.

[80] Law CW, Alhamdoosh M, Su S, Dong X, Tian L, Smyth GK, Ritchie ME (2016). RNA-seq analysis is easy as 1–2-3 with limma, Glimma and edgeR. F1000Res.

[81] Anders S, Huber W (2010). Differential expression analysis for sequence count data. Genome Biol.

[82] Emms DM, Kelly S (2015). OrthoFinder: solving fundamental biases in whole genome comparisons dramatically improves orthogroup inference accuracy. Genome Biol.

[83] Hellens RP, Allan AC, Friel EN, Bolitho K, Grafton K, Templeton MD, Karunairetnam S, Gleave AP, Laing WA (2005). Transient expression vectors for functional genomics, quantification of promoter activity and RNA silencing in plants. Plant Methods.

