# Supplementary figures and images for: Comparative transcriptome analyses of fruit development among pears, peaches, and strawberries provide new insights into single sigmoid patterns
Source: BMC Plant Biol. 2020 Mar 6;20:108. doi: 10.1186/s12870-020-2317-6 (PMC7060524; doi:10.1186/s12870-020-2317-6)

**Cv. Housui**

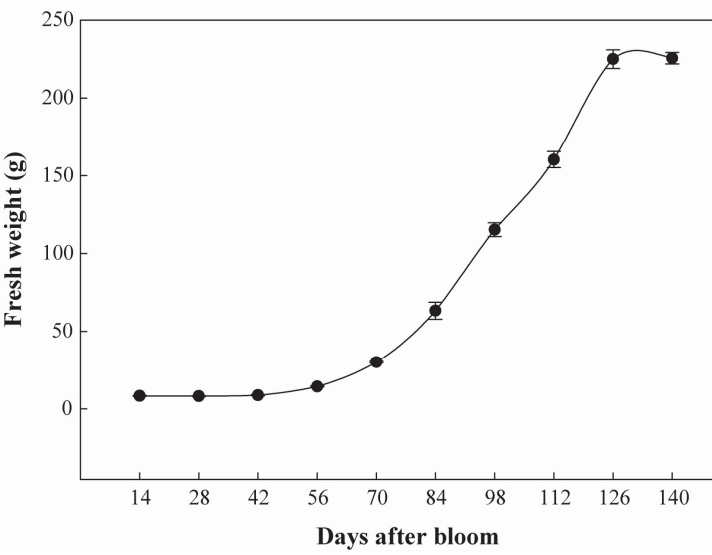

**Cv. Cuiguan**

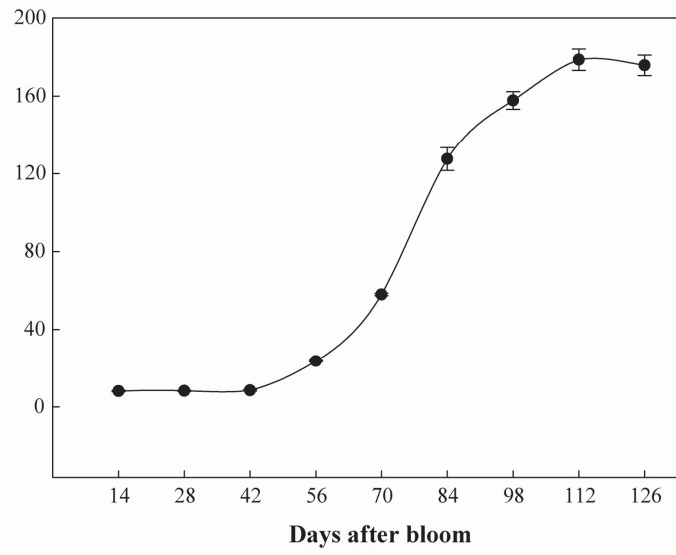

**Cv. Xueqing**

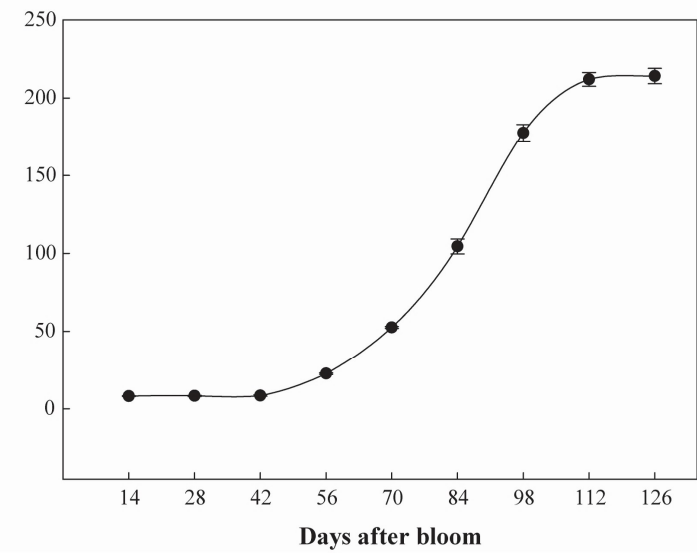

Supplement: Supplementary file 1 — Additional file 1: Figure S1. Measurements of fruit weight in Cvs. ‘Housui’, ‘Cuiguan’ and ‘Xueqing’. [file 12870_2020_2317_MOESM1_ESM.pdf]

**A**

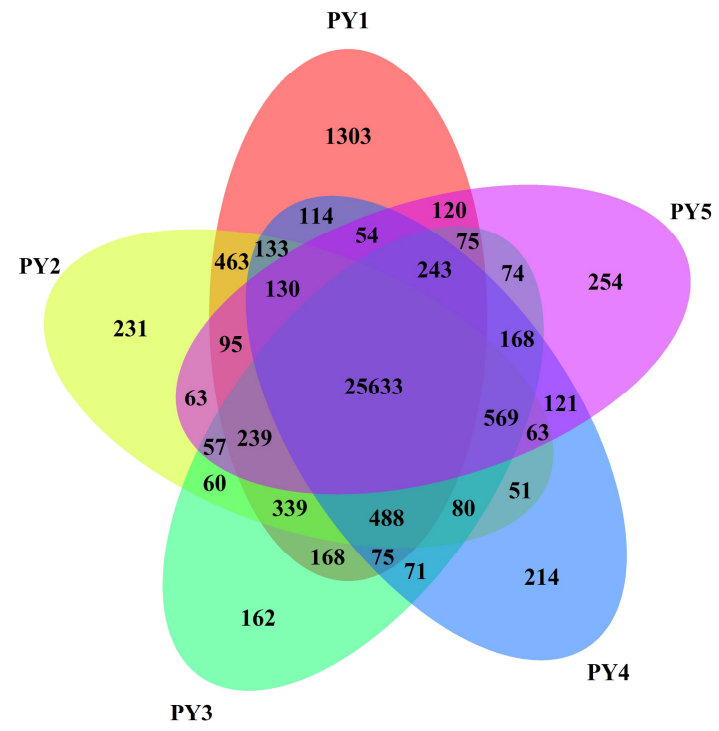

**B**

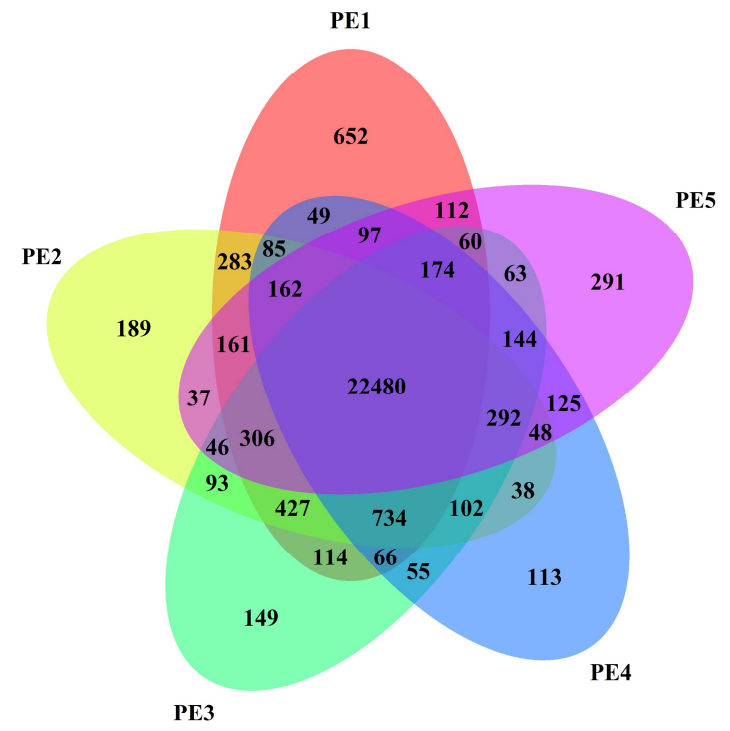

**C**

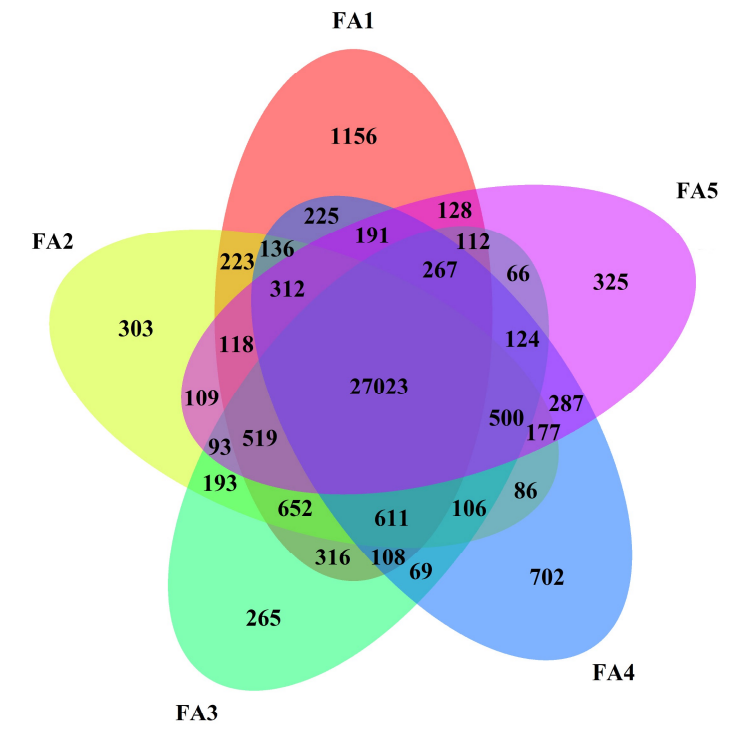

Supplement: Supplementary file 2 — Additional file 2: Figure S2. Commonly and specially expressed genes in five period of three fruit species. [file 12870_2020_2317_MOESM2_ESM.pdf]

A

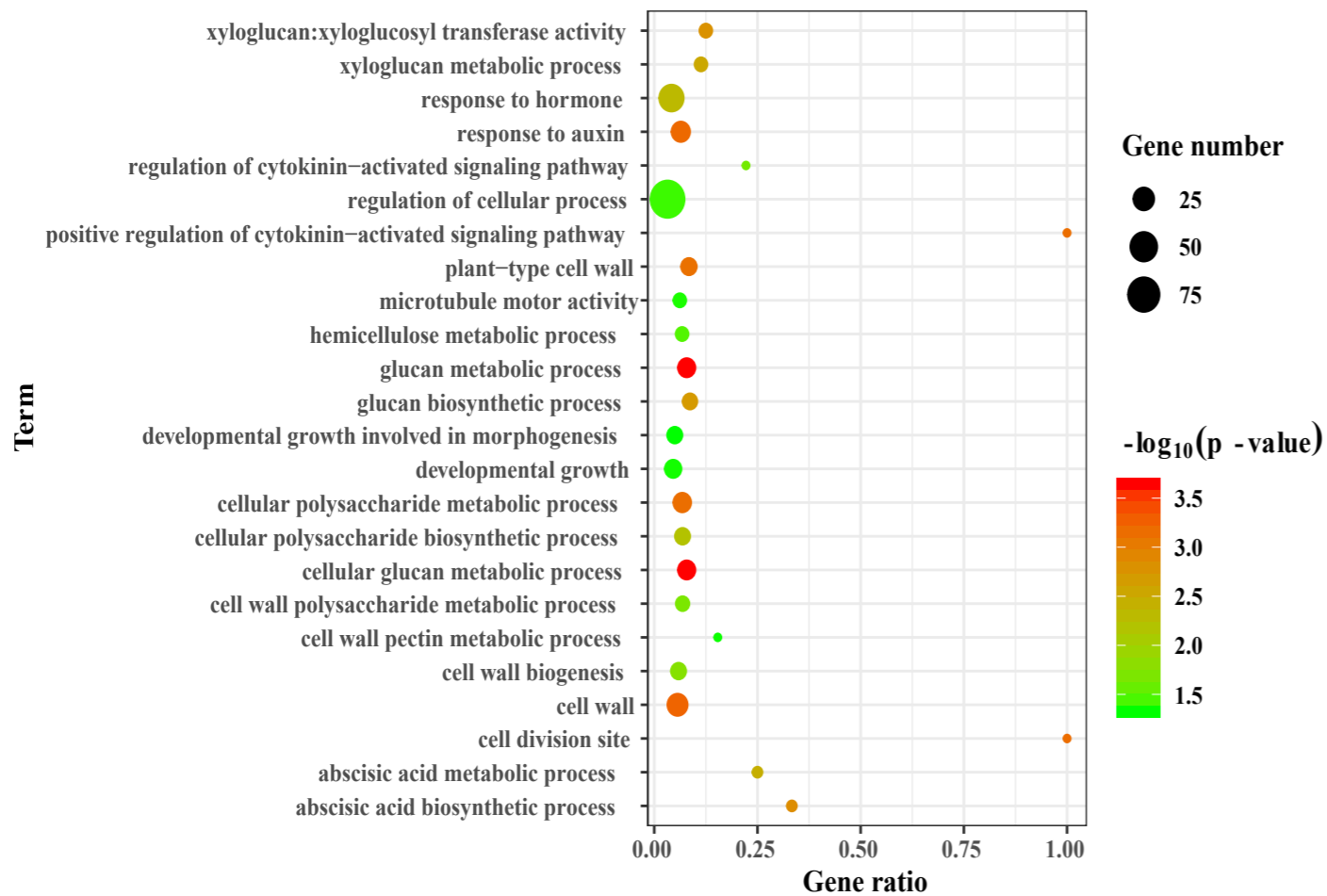

B

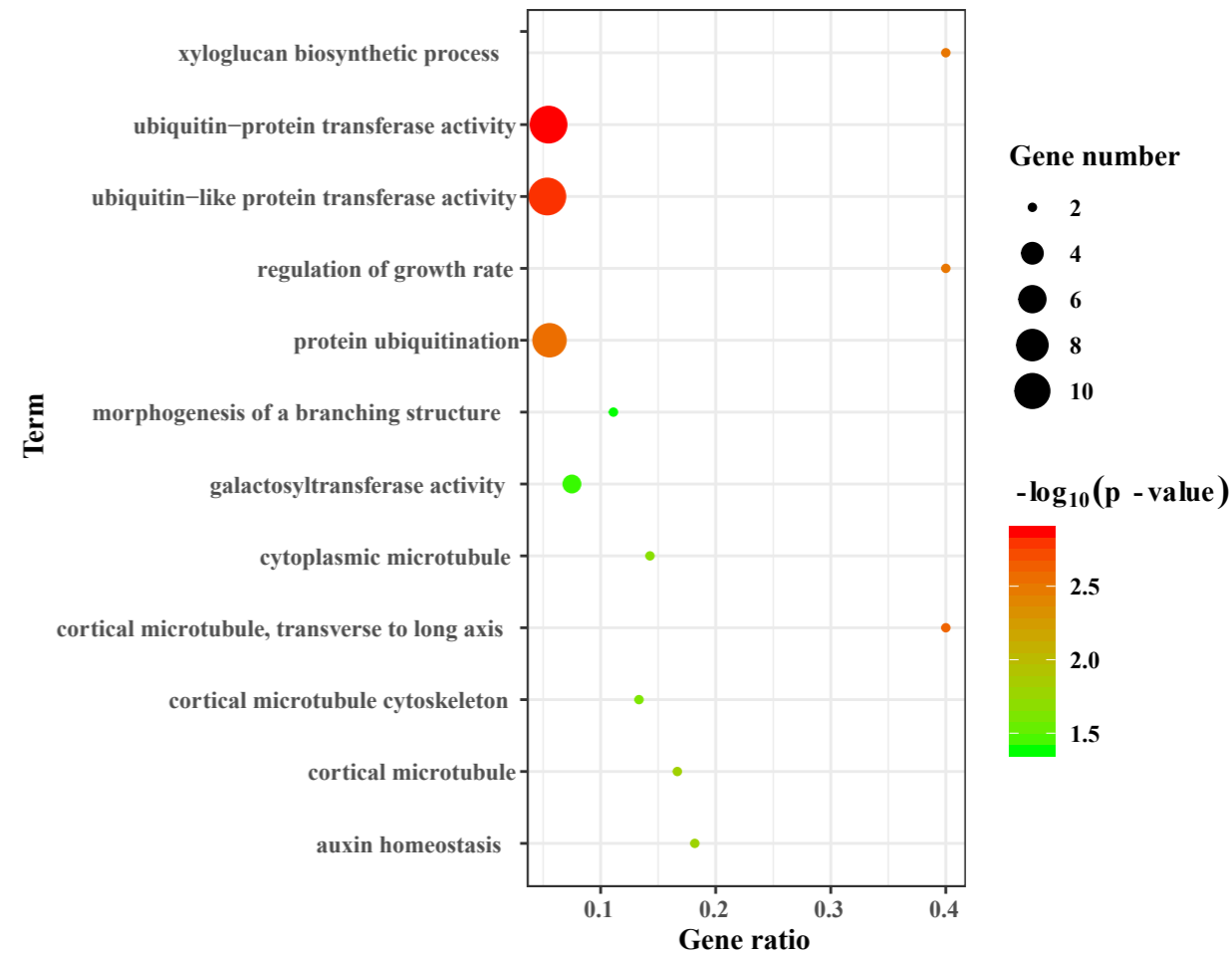

Supplement: Supplementary file 3 — Additional file 3: Figure S3. GO enrichment of candidate genes identified in Model I and Model II. [file 12870_2020_2317_MOESM3_ESM.pdf]

1 cm

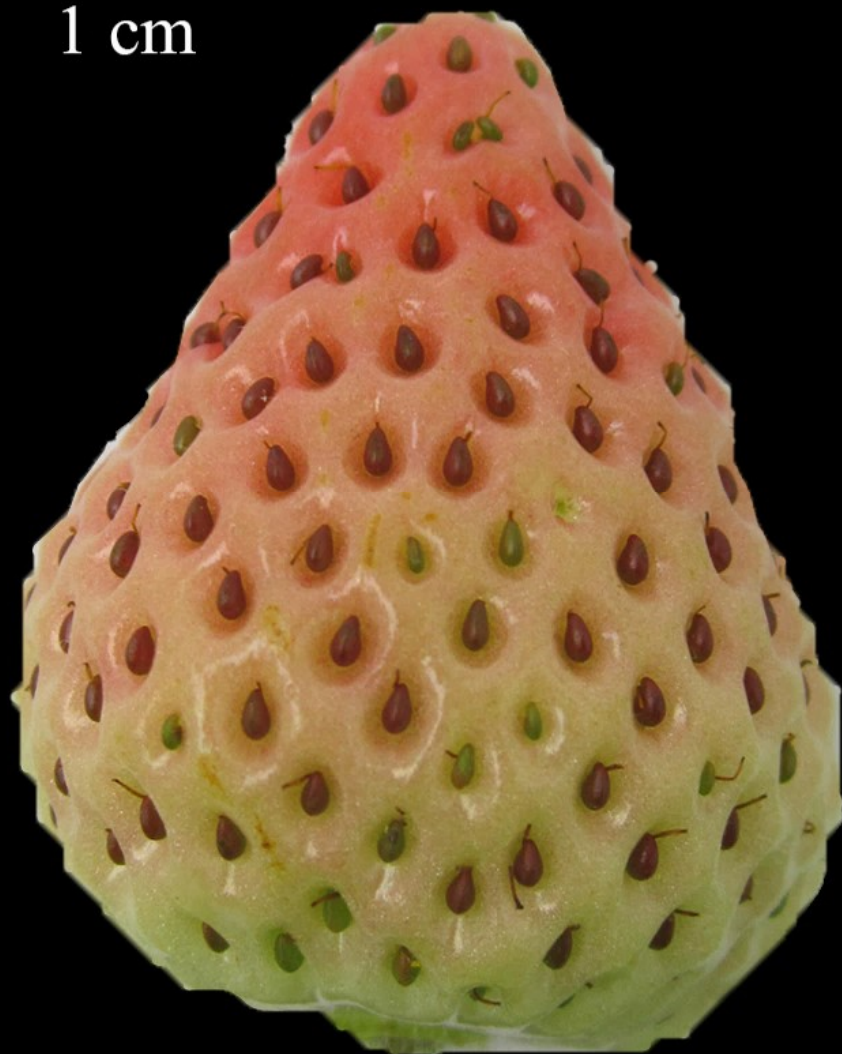

31DAF

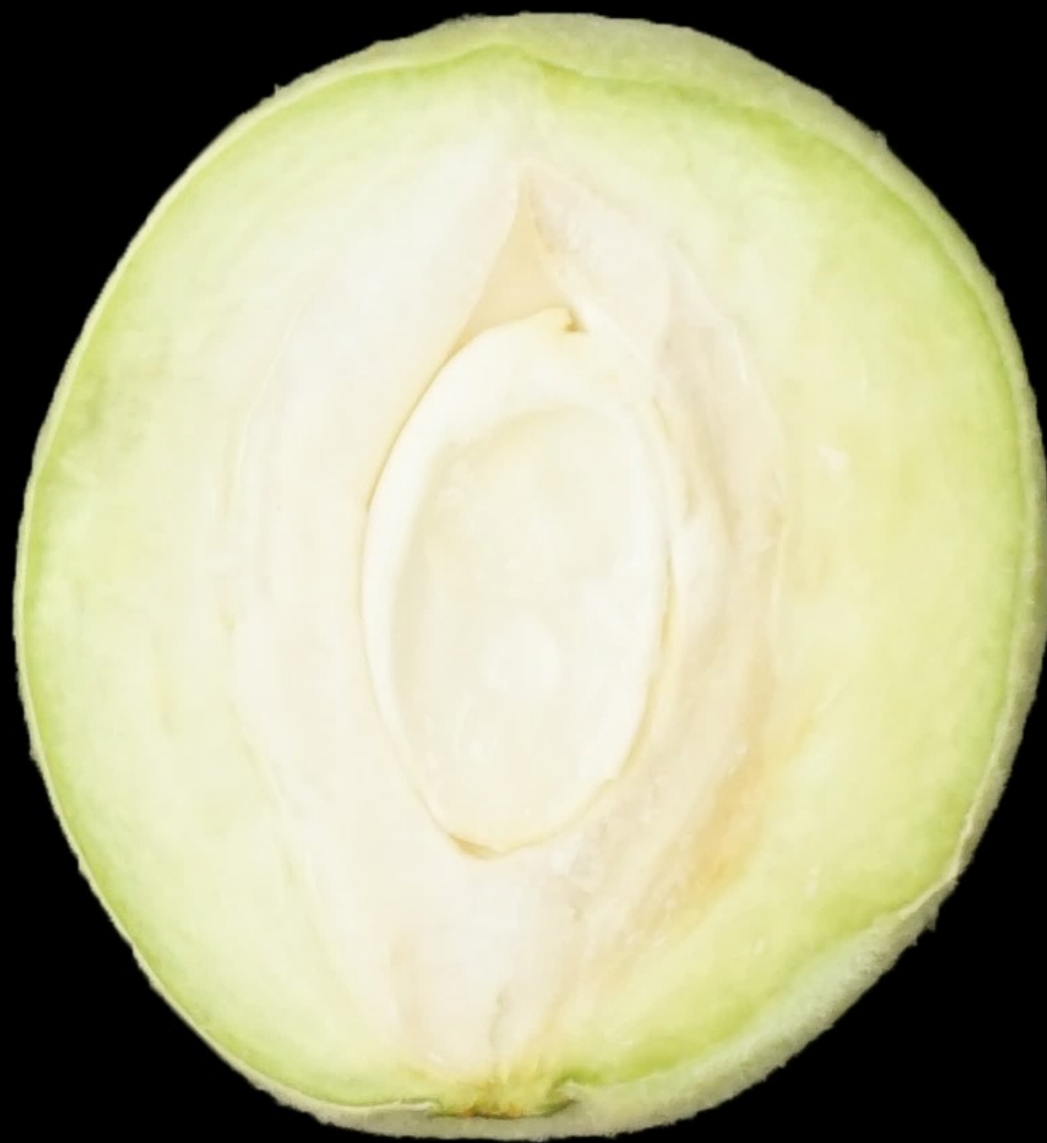

56DAF

Supplement: Supplementary file 5 — Additional file 5: Figure S5. The pictures of strawberries and peaches in the intervals. [file 12870_2020_2317_MOESM5_ESM.pdf]
