# Supplementary material for: Comparative transcriptome analyses of fruit development among pears, peaches, and strawberries provide new insights into single sigmoid patterns
Source: BMC Plant Biol. 2020 Mar 6;20:108. doi: 10.1186/s12870-020-2317-6 (PMC7060524; doi:10.1186/s12870-020-2317-6)
Supplement: Supplementary file 4 — Additional file 4: Figure S4. Expression profiles of the differential expressed genes related to fruit enlargements in pears, peaches and strawberries, respectively. [file 12870_2020_2317_MOESM4_ESM.pdf]

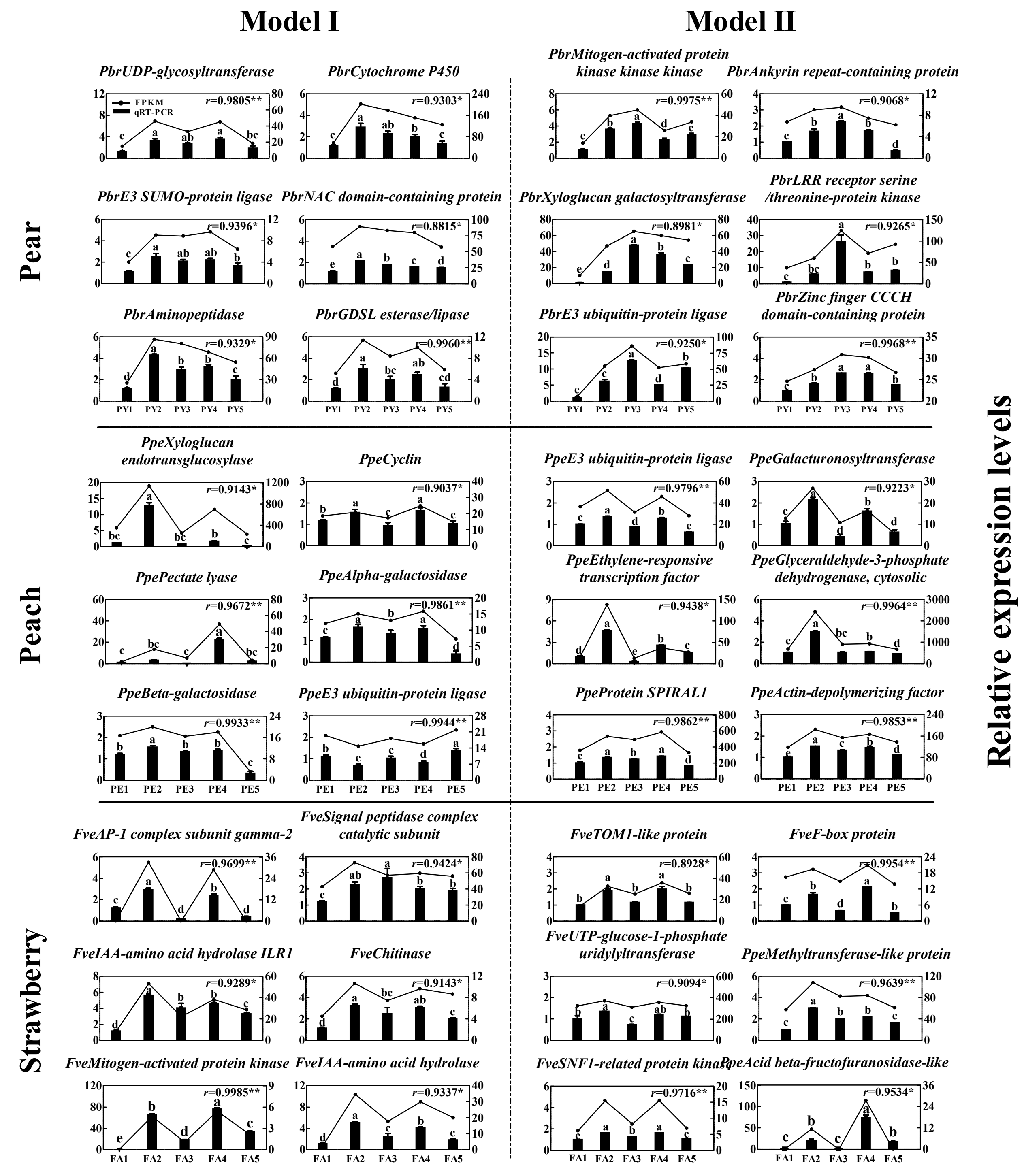

Model I
Model II
Pear
Peach
Strawberry
Relative expression levels
FPKM
qRT-PCR
PbrUDP-glycosyltransferase
r=0.9805**
PbrCytochrome P450
r=0.9303*
PbrMitogen-activated protein kinase kinase kinase
r=0.9975**
PbrAnkyrin repeat-containing protein
r=0.9068*
PbrE3 SUMO-protein ligase
r=0.9396*
PbrNAC domain-containing protein
r=0.8815*
PbrXyloglucan galactosyltransferase
r=0.8981*
PbrLRR receptor serine /threonine-protein kinase
r=0.9265*
PbrAminopeptidase
r=0.9329*
PbrGDSL esterase/lipase
r=0.9960**
PbrE3 ubiquitin-protein ligase
r=0.9250*
PbrZinc finger CCCH domain-containing protein
r=0.9968**
PY1 PY2 PY3 PY4 PY5
PpeXyloglucan endotransglucosylase
r=0.9143*
PpeCyclin
r=0.9037*
PpeE3 ubiquitin-protein ligase
r=0.9796**
PpeGalacturonosyltransferase
r=0.9223*
PpePectate lyase
r=0.9672**
PpeAlpha-galactosidase
r=0.9861**
PpeEthylene-responsive transcription factor
r=0.9438*
PpeGlyceraldehyde-3-phosphate dehydrogenase, cytosolic
r=0.9964**
PpeBeta-galactosidase
r=0.9933**
PpeE3 ubiquitin-protein ligase
r=0.9944**
PpeProtein SPIRAL1
r=0.9862**
PpeActin-depolymerizing factor
r=0.9853**
PE1 PE2 PE3 PE4 PE5
FveAP-1 complex subunit gamma-2
r=0.9699**
FveSignal peptidase complex catalytic subunit
r=0.9424*
FveTOM1-like protein
r=0.8928*
FveF-box protein
r=0.9954**
FveIAA-amino acid hydrolase ILR1
r=0.9289*
FveChitinase
r=0.9143*
FveUTP-glucose-1-phosphate uridylyltransferase
r=0.9094*
PpeMethyltransferase-like protein
r=0.9639**
FveMitogen-activated protein kinase
r=0.9985**
FveIAA-amino acid hydrolase
r=0.9337*
FveSNF1-related protein kinase
r=0.9716**
PpeAcid beta-fructofuranosidase-like
r=0.9534*
FA1 FA2 FA3 FA4 FA5
